# Ammonium triggered the response mechanism of lysine crotonylome in tea plants

**DOI:** 10.1186/s12864-019-5716-z

**Published:** 2019-05-06

**Authors:** Jianhao Sun, Chen Qiu, Wenjun Qian, Yu Wang, Litao Sun, Yusheng Li, Zhaotang Ding

**Affiliations:** 1 Tea Research Institute, Qingdao Agricultural University, Qingdao, 266109 Shandong China; 2 Fruit and Tea Technology Extension Station, Jinan, 250000 Shandong China

**Keywords:** *Camellia sinensis* L., Lysine crotonylation, Ammonium deficiency/resupply, Primary metabolism, Enzymatic activity

## Abstract

**Background:**

Lysine crotonylation, as a novel evolutionarily conserved type of post-translational modifications, is ubiquitous and essential in cell biology. However, its functions in tea plants are largely unknown, and the full functions of lysine crotonylated proteins of tea plants in nitrogen absorption and assimilation remains unclear. Our study attempts to describe the global profiling of nonhistone lysine crotonylation in tea leaves and to explore how ammonium (NH_4_^+^) triggers the response mechanism of lysine crotonylome in tea plants.

**Results:**

Here, we performed the global analysis of crotonylome in tea leaves under NH_4_^+^ deficiency/resupply using high-resolution LC-MS/MS coupled with highly sensitive immune-antibody. A total of 2288 lysine crotonylation sites on 971 proteins were identified, of which contained in 15 types of crotonylated motifs. Most of crotonylated proteins were located in chloroplast (37%) and cytoplasm (33%). Compared with NH_4_^+^ deficiency, 120 and 151 crotonylated proteins were significantly changed at 3 h and 3 days of NH_4_^+^ resupply, respectively. Bioinformatics analysis showed that differentially expressed crotonylated proteins participated in diverse biological processes such as photosynthesis (PsbO, PsbP, PsbQ, Pbs27, PsaN, PsaF, FNR and ATPase), carbon fixation (rbcs, rbcl, TK, ALDO, PGK and PRK) and amino acid metabolism (SGAT, GGAT2, SHMT4 and GDC), suggesting that lysine crotonylation played important roles in these processes. Moreover, the protein-protein interaction analysis revealed that the interactions of identified crotonylated proteins diversely involved in photosynthesis, carbon fixation and amino acid metabolism. Interestingly, a large number of enzymes were crotonylated, such as Rubisco, TK, SGAT and GGAT, and their activities and crotonylation levels changed significantly by sensing ammonium, indicating a potential function of crotonylation in the regulation of enzyme activities.

**Conclusions:**

The results indicated that the crotonylated proteins had a profound influence on metabolic process of tea leaves in response to NH_4_^+^ deficiency/resupply, which mainly involved in diverse aspects of primary metabolic processes by sensing NH_4_^+^, especially in photosynthesis, carbon fixation and amino acid metabolism. The data might serve as important resources for exploring the roles of lysine crotonylation in N metabolism of tea plants. Data were available via ProteomeXchange with identifier PXD011610.

**Electronic supplementary material:**

The online version of this article (10.1186/s12864-019-5716-z) contains supplementary material, which is available to authorized users.

## Background

Ammonium (NH_4_^+^) and nitrate (NO_3_^−^) are the major sources of nitrogen (N) in higher plants. As a kind of beverage leafy crop, tea plant (*Camellia sinensis* L.) preferred NH_4_^+^ over NO_3_^−^ [1, 2]. Compared with the NH_4_^+^ supply, the contents of chlorophyll and biomass were more lower in tea plants when NO_3_^−^ was supplied as the sole nitrogen source [3]. Compared with NO_3_^−^ supply, the biosynthesis of free amino acids and catechins was more effective in tea plants under NH_4_^+^ supply, resulting from expression of N transporter genes [4]. NH_4_^+^ deficiency could lead to clearly reduce the accumulation of amino acid, while NH_4_^+^ supply could improve the status of carbohydrate in tea plants [1, 4]. Moreover, previous study in our laboratory showed that the levels of lysine acetylation in tea leaves changed dynamically under NH_4_^+^ resupply. And lysine acetylated proteins might regulate photosynthesis, glycolysis and secondary metabolism in tea leaves after NH_4_^+^ resupply [5]. However, the global profiling of lysine crotonylated proteins in tea plants in response to NH_4_^+^ resupply remains largely unknown.

Post-translational modifications (PTMs) of histone were well-known for its critical roles in cellular pathways, which could change the physicochemical properties of proteins and affect their activity and stability [6, 7]. As a novel evolutionarily conserved type of PTMs, the histone crotonylation level could be regulated by cellular concentration of crotonyl-CoA [8]. The previous reports demonstrated that a subset of genes could be differentially regulated by histone crotonylation, and selective histone decrotonylation could repress the global transcription of mouse embryonic stem cells to some extent [9]. Recently, the global profiling of crotonylation has been reported in tobacco and rice [10, 11], which revealed that crotonylation was correlated with signal transduction and cellular physiology. However, there have not been systematically reported about the dynamic viewing of nonhistone lysine crotonylation responding to N, especially ammonium. In order to explore whether the crotonylation is connected with NH_4_^+^ resupply and to obtain a comprehensive characterization of lysine crotonylation in tea leaves, we adopted an integrated system using the peptide prefractionation, immunoaffinity enrichment, and coupling with highly sensitive mass spectrometry combined with affinity purification analysis. We examined the crotonylated proteins in tea leaves under NH_4_^+^ deficiency/resupply and analyzed the Gene Ontology (GO), Kyoto Encyclopedia of Genes and Genomes (KEGG) and protein-protein interaction (PPI) of these proteins. This research not only greatly extended the list of crotonylated proteins in tea plants but also paved the way for exploring the roles of crotonylated proteins in the utilization of N in tea plants.

## Methods

### Plant materials and growth conditions

The 1-year-old seedlings of the tea plants cultivar ‘QN3’ planted in pots at the greenhouse of the Tea Research Institute, Qingdao Agricultural University in Shandong Province of China (36°19′ N, 120°23′ E, 54.88 m above the sea level), were used to Hydroponics. For hydroponics, the tea plants were suspended in a hydroponic nutrient solution (Additional file 1). The growth conditions were set as follows: temperature, 25 ± 1 °C /15 ± 1 °C (14 h day/10 h night); lighting, 260–280 μM•m^− 2^•s^− 1^ photon flux densities; and humidity, 75 ± 5% relative humidity. After hydroponic seedlings were grown in the greenhouse for two weeks to recover, which used to perform the NH_4_^+^ deficiency/resupply experiments. Firstly, hydroponic seedlings were transferred to NH_4_^+^ deficiency nutrient solution ((NH_4_)_2_SO_4_ deleted) for 14 days (tea plants of NH_4_^+^ deficiency). Then, these seedlings were retransferred to NH_4_^+^ resupply nutrient solution ((NH_4_)_2_SO_4_ added) for 3 days. Finally, the third and/or fourth mature leaves from the terminal bud were sampled at NH_4_^+^ deficiency (DN), 3 h (3hN) and 3 days (3dN) of NH_4_^+^ resupply. All samples were quickly frozen in liquid nitrogen and stored at − 80 °C for further study. Three biological replicates were performed for each sampling time point.

### Physiological determinations

For physiological experiments, more than ten plants were harvested and pooled for each treatment group at DN, 3hN and 3dN. And the leaves were collected three times as biological replicates. The nitrogen content (*NC*) of tea leaves was measured after Kjeldahl digestion. The chlorophyll content (*CC*) of samples was measured as described by Lichtenthaler et al [12]*.* The measurement of obtain maximum photochemical quantum yield of PS II (*Fv/Fm*) referred to Zheng et al. [13]. Samples were analyzed for the contents of free amino acids (*AA*), and measurement followed the Sate Standard of China for tea content determination recorded as: GB 8314–87.

### Western blot

The samples of tea leaves that in been grown in the presence of DN, 3hN and 3dN, were performed by western blotting (WB) analysis based on a previously described method [14]. For western blot, protein was diluted with SDS loading buffer, and 30 μg protein of each sample was separated by 12% SDS-PAGE and electro-blotted onto PVDF. First antibody and second antibody were probed using Anti-crotonyllysine Antibody (PTM-502, PTM Biolabs, Hangzhou, China) in the 1:1000 dilution and HRP AffiniPure Goat Anti-Rabbit IgG (31,430, Thermo Fisher Scientific, Waltham, USA) in 1:10000 dilution, respectively.

### Protein extraction and digestion

The protein extraction and digestion of the samples were following by previous method [15]. And then the protein solution was reduced with 5 mM dithiothreitol for 30 min at 56 °C and alkylated with 11 mM iodoacetamide for 15 min at room temperature in darkness. The protein sample was then diluted by adding 100 mM NH_4_HCO_3_ to urea concentration less than 2 M. Finally, trypsin (Promega, Madison, WI, USA) was added at 1:50 trypsin-to-protein mass ratio for 12 h and 1:100 trypsin-to-protein mass ratio for a second 4 h-digestion. And the more detailed information was shown in Additional file 2.

### HPLC fractionation and affinity enrichment

For global proteome analysis**,** the tryptic peptides were fractionated into fractions by high pH reverse-phase HPLC using Agilent 300 Extend C18 column (5 μm particles, 4.6 mm ID, 250 mm length). Briefly, peptides were first separated with a gradient of 8 to 32% acetonitrile (pH 9.0, Fisher Chemical) over 60 min into 60 fractions. Then, the peptides were combined into four fractions and dried by vacuum centrifuging. 200 μg peptides were used for HPLC fractionation in this process.

To enrich crotonylated peptides, tryptic peptides dissolved in NETN buffer (100 Mm NaCl, 1 mM EDTA, 50 mM Tris-HCl, 0.5% NP-40, pH 8.0) were incubated with pre-washed antibody beads (PTM-402, PTM Biolabs, Hangzhou, China) at 4 °C overnight with gentle shaking. Then the beads were washed four times with NETN buffer and twice with H_2_O. The bound peptides were eluted from the beads with 0.1% trifluoroacetic acid (Sigma, Saint Louis, USA). Finally, the eluted fractions were combined and vacuum-dried. For LC-MS/MS analysis, the resulting peptides were desalted with C18 ZipTips (Millipore, Saint Louis, USA) according to the manufacturer’s instructions, followed by LC-MS/MS analysis. In this process, 1.5 mg peptides were used for each affinity enrichment.

### LC-MS/MS analysis

The tryptic peptides were dissolved in solvent A (water on 0.1% formic acid) and directly loaded onto a home-made reversed-phase analytical column (15-cm length, 75 μm i.d.). For global proteome analysis, the gradient of was comprised of an increase from 6 to 23% solvent B (0.1% formic acid in 90% acetonitrile) over 40 min, 23 to 35% in 14 min and climbing to 80% in 3 min then holding at 80% for the last 3 min, all at a constant flow rate of 400 nL/min on an EASY-nLC 1000 UPLC system (Thermo Fisher Scientific, Waltham, USA). For crotonylome analysis, the gradient of was comprised of an increase from 7 to 25% solvent B (0.1% formic acid in 90% acetonitrile) over 38 min, 25 to 40% in 14 min and climbing to 80% in 4 min then holding at 80% for the last 4 min, all at a constant flow rate of 700 nL/min on an EASY-nLC 1000 UPLC system.

The peptides were subjected to NSI (neutral spray ionization) source followed by tandem mass spectrometry (MS/MS) in Q Exactive™ Plus (Thermo Fisher Scientific, Waltham, USA) coupled online to the UPLC. The electrospray voltage applied was 2.0 Kv for crotonylome analysis or 2.1 Kv for global proteome analysis. The m/z scan range was 350 to 1800 for full scan, and intact peptides were detected in the Orbitrap at a resolution of 70,000. Peptides were then selected for MS/MS using NCE setting as 28 and the fragments were detected in the Orbitrap at a resolution of 17,500. In order to improve the efficiency of mass spectrometry, automatic gain control (AGC) was set at 5E4, signal threshold was set at 10000 ions/s for crotonylome analysis or 20,000 ions/s for global proteome analysis, the maximum injection time was set at 200 ms for crotonylome analysis or 100 ms for global proteome analysis, and the dynamic exclusion time of tandem mass spectrometry was set at 15 s for crotonylome analysis or 30 s for global proteome analysis.

### Database search and bioinformatic analysis

For protein quantification of global proteome and crotonylome, the resulting MS/MS data were processed using MaxQuant search engine (v.1.5.2.8). Tandem mass spectra were searched against *Camellia sinensis* database (36,951 sequences, http://www.plantkingdomgdb.com/tea_tree/) concatenated with reverse decoy database. Trypsin/P was specified as cleavage enzyme allowing up to 4 missing cleavages for lysine crotonylome, and 2 missing cleavages for global proteome. The mass tolerance for precursor ions was set as 20 ppm in First search and 5 ppm in Main search, and the mass tolerance for fragment ions was set as 0.02 Da. For global proteome analysis, carbamidomethyl on Cys was specified as fixed modification, oxidation on Met and acetylation on the protein N-terminus was specified as variable modifications. For crotonylome analysis, carbamidomethyl on Cys for fixed modification and oxidation on Met, crotonylation on lysine and acetylation on the protein N-terminus for variable modifications. And then, Label-free quantification method was LFQ, FDR was adjusted to < 1% and minimum score for peptides or modified peptides were set > 40.

Soft motif-x (http://motif-x.med.harvard.edu/) was used to analyze the model of sequences with amino acids in specific positions of modify-21-mers (10 amino acids upstream and downstream of the site) in all protein sequences. The subcellular localization was performed by wolfpsort (http://www.genscript.com/wolf-psort.html). The GO annotation and enrichment analyses were done by UniProt-GOA database (http://www.ebi.ac.uk/GOA/). KEGG database was adopted for the enrichment of pathways by functional annotation tool of DAVID against the background of *Camellia sinensis*. To perform a PPI network analysis, the STRING database (http://string-db.org/) was used and then functional protein interaction networks were visualized by using Cytoscape (v.3.7.0).

### Enzyme assays

The activity of Rubisco, TK, PRK, SGAT, GGAT and SHMT were measured at DN, 3hN and 3dN in tea leaves, using ELISA kit from Jiangsu Meimian industrial Co. Ltd. The enzyme activity was tested using ELISA kit from Jiangsu Meimian industrial Co. Ltd. in 50 mg sample of each enzyme. Finally, the reaction was terminated by the addition of a sulphuric acid solution and the color change was measured spectrophotometrically at a wavelength of 450 nm. Each treatment was designed with three replicates randomly. The more detail information was shown in Additional file 3.

## Results

### Physiological characterization of tea leaves after NH_4_^+^ resupply

To describe the major physiological changes of tea plants in varying NH_4_^+^ resupply, we measured the *NC*, CC, *Fv/Fm* and *AA* under NH_4_^+^ deficiency/resupply (Additional file 4: Figure S1). We found that *NC*, CC, *Fv/Fm* and *AA* at DN were all lower than those of NH_4_^+^ resupply, and they showed upward trends with the NH_4_^+^ resupply prolonged (3hN and 3dN). These results indicated that NH_4_^+^ could affect the physiological property of tea leaves.

### WB analysis of tea leaves after NH_4_^+^ resupply

**Fig. 1:**
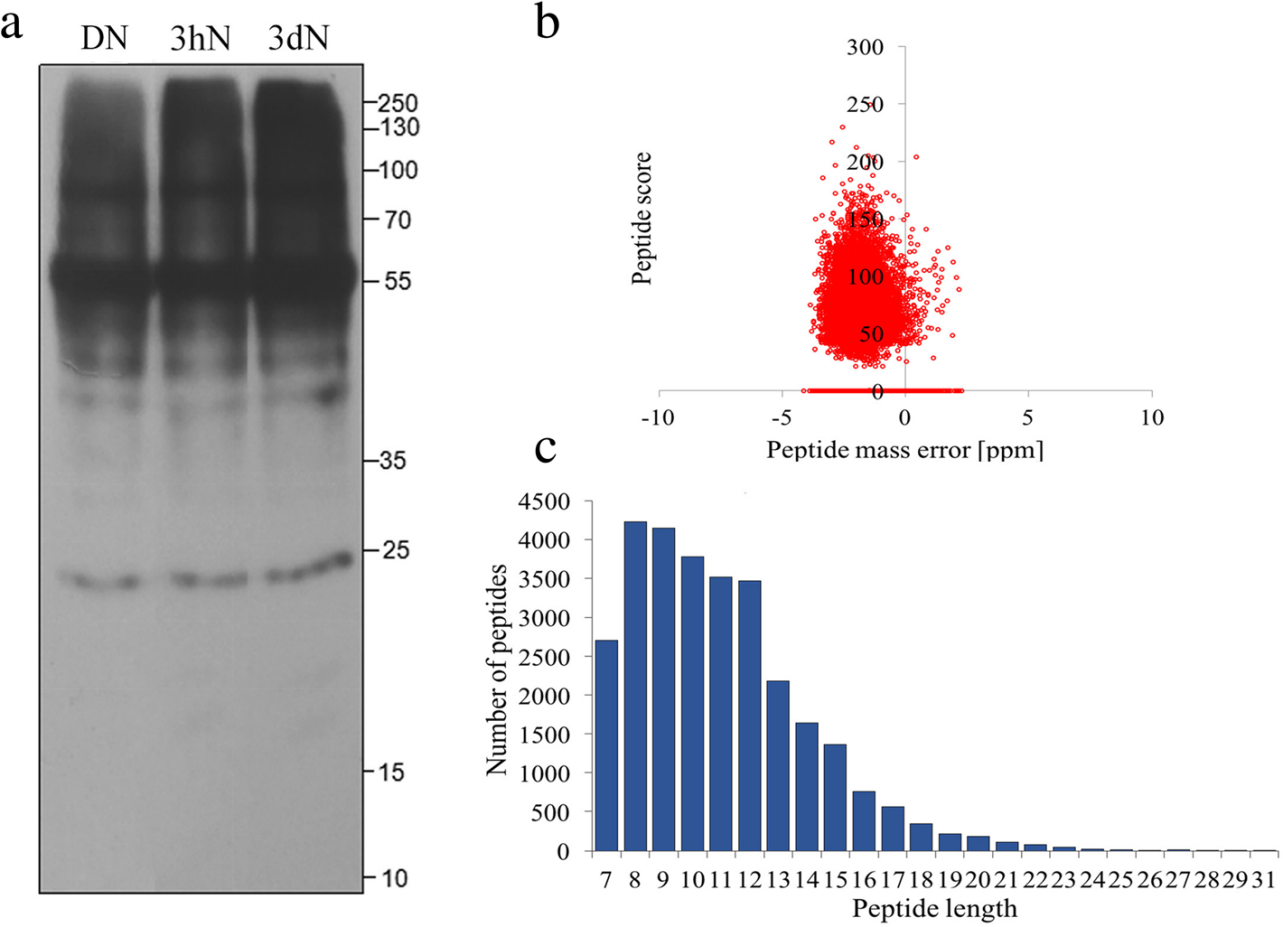
The WB analysis and proteome-wide identification of lysine crotonylation sites in tea leaves. **a** WB analysis of the total protein content of tea leaves showing duplicates at three time-points after NH_4_^+^ resupply. **b** The mass error distributions of crotonylation profiles. **c** The peptide length distributions of crotonylation profiles

In order to test whether there was crotonylation in proteins of tea leaves after NH_4_^+^ resupply, we performed SDS-PAGE and WB analysis. As a quantitative control in three samples, the results of SDS-PAGE showed that the amount of proteins added to the three samples was consistent (Additional file 4: Figure S2). And then, the results of WB showed that proteins in tea leaves were widely crotonylated, and crotonylation levels showed changes between each time point (Fig. 1a), suggesting that lysine crotonylation underwent significantly dynamic changes in response to NH_4_^+^ resupply.

### Detection of lysine crotonylome and global proteome in tea leaves

In this study, we combined antibody against crotonylated lysine, LC − MS/MS and intensive bioinformatics for comprehensive study of lysine crotonylome in tea leaves upon NH_4_^+^ treatment. In order to validate the MS data, we firstly checked the mass error of the identified peptides. The distribution of mass errors was near zero, and most errors were less than 4 ppm, indicating the accuracy of the MS data (Fig. 1b). The lengths of most identified peptides were in the range of 7 to 17 amino acids, which were consistent with the property of tryptic peptides, indicating that the sample preparation achieved a reasonable standard (Fig. 1c). Each treatment was designed with three biological replicates. As a result, a total of 2260 modified peptides were identified on 971 proteins, containing 2288 crotonylation sites (Additional file 5). Among of them, the majority of crotonylated proteins (698, 71.9%) identified contained only one or two crotonylation sites (Additional file 4: Figure S3). Fifty representative LC-MS/MS spectra of crotonylated peptides were shown in Additional file 6. Furthermore, global proteome data for normalization were also collected with identified 5312 protein groups (Additional file 7).

### Functional classification and motif analysis of the lysine crotonylome in tea leaves

To better understand the function of proteins in crotonylome and global proteome, we conducted their functional classification. The results of subcellular localization revealed that a majority of crotonylated proteins were mainly located in chloroplast (36.8%), cytoplasm (33.0%) and nucleus (13.7%), suggesting that the crotonylated proteins distributed broadly in tea leaves (Additional file 8: Fig. 2a). The subcellular localization of global proteome showed that the proteins were mainly located in chloroplast (34.7%), cytoplasm (30.3%) and nucleus (17.8%). Then, the analysis of molecular functions showed that crotonylated proteins and global proteins presented a similar pattern, and the vast majority of proteins participated in binding and catalytic activity (Additional file 8: Fig. 2b). And the percentage of crotonylome and global proteome in each category was also very close. Based on the results, we found that the subcellular localization and molecular functions between the crotonylome and global proteome have no significant difference.

**Fig. 2:**
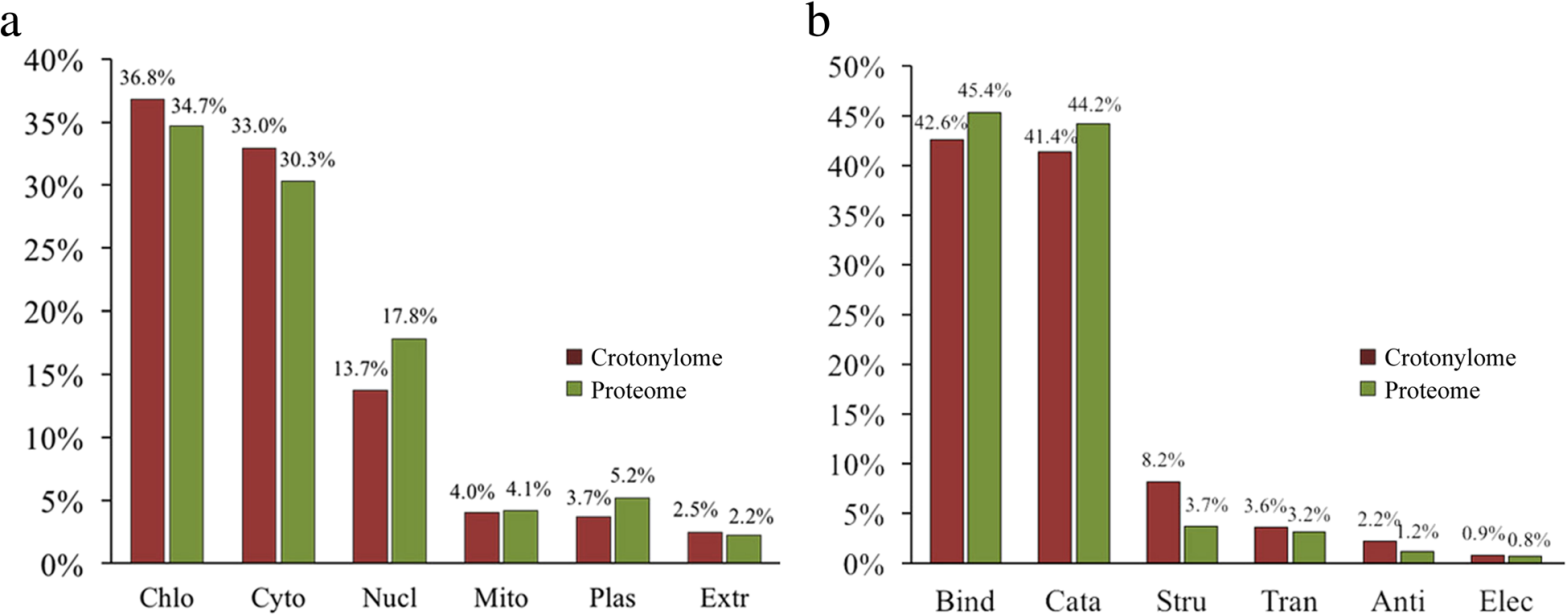
Functional classification of lysine crotonylome compared to global proteome. **a** Subcellular localization of lysine crotonylome compared to global proteome. **b** Molecular function of lysine crotonylome compared to global proteome. Abbreviation: Chlo for chloroplast, Cyto for cytoplasm, Nucl for nucleus, Mito for mitochondria, Plas for plasma membrane, Extr for extracellular, Bind for binding, Cata for catalytic activity, Stru for structural molecule activity, Tran for transporter activity, Anti for antioxidant activity, and Elec for electron carrier activity

**Fig. 3:**
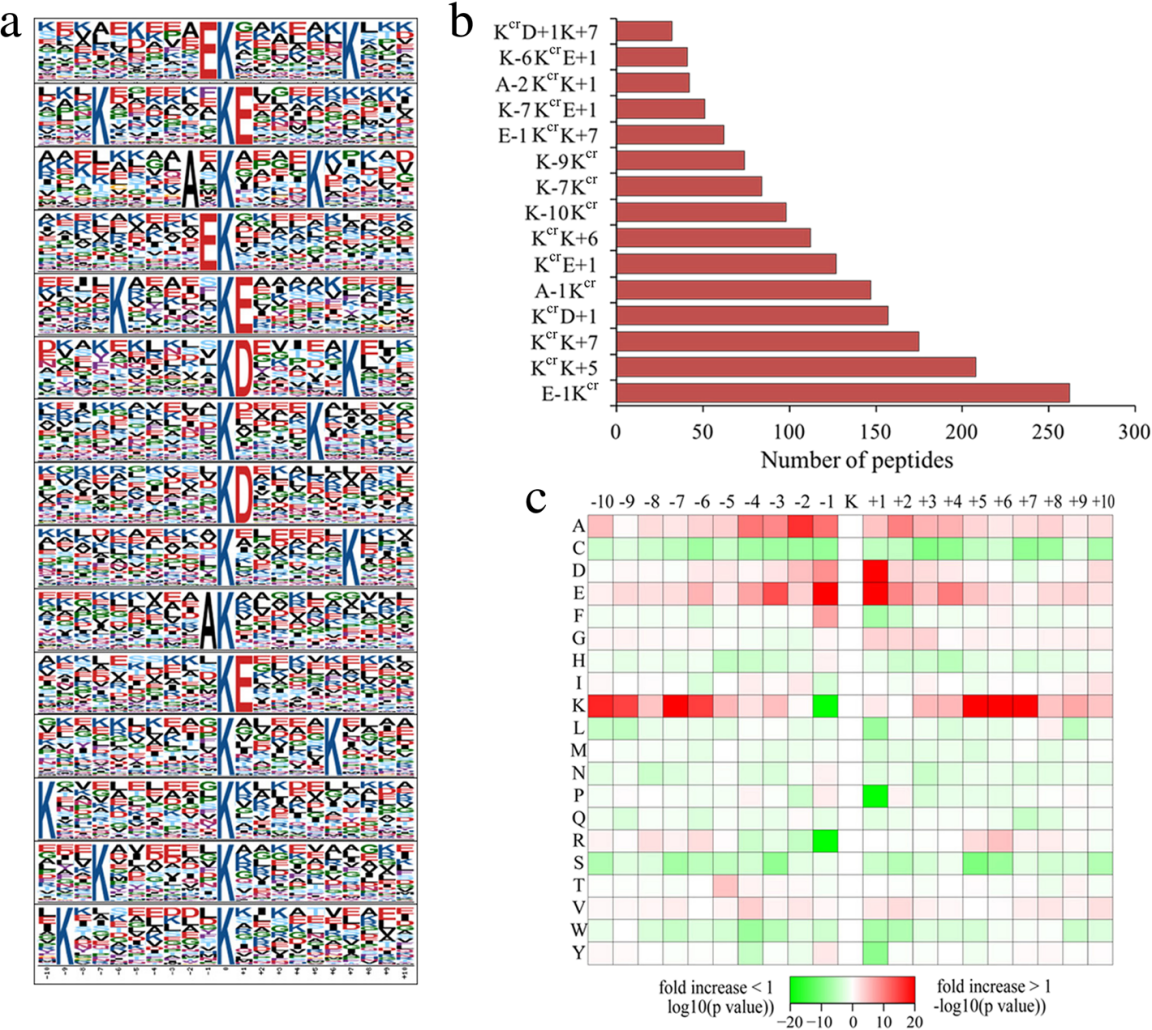
Motif analysis of lysine crotonylated peptides. **a** Crotonylated sequence motifs and conservation of crotonylation sites. The 0 position K refers to the crotonylation sites. **b** Number of identified peptides containing crotonylation in each motif. **c** Heatmap of the amino acid compositions of the crotonylation sites showing the frequency of the different of amino acids around the crotonylation. “+ 1” and “-1” represent the position around the crotonylation

To determine whether there were any specific amino acid biases adjacent to crotonylation sites, we investigated the sequence of all identified crotonylated peptides with the motif-x program (Additional file 9). As a result, the amino acid sequences were classified into 15 conserved motifs, including E-1K^cr^, K^cr^D + 1 K + 7, K^cr^K + 5, K^cr^D + 7, A-1K^cr^, K^cr^E+ 1/+ 6, K-10/− 9/−7K^cr^, E-1K^cr^K + 7, K-7/−6K^cr^E+ 1, A-2K^cr^K + 1 (Fig. 3a). Among them, E-1K^cr^, K^cr^D + 1, A-1K^cr^, K^cr^D + 1 and K^cr^E+ 1 have been reported as crotonylation motifs [10, 16], while the others were first reported in our study. In addition, we found that the abundances of E-1K^cr^, E-1K^cr^, K^cr^K + 7 and K^cr^D + 1 were comparatively higher than the other 11 conserved motifs (Fig. 3b). In accordance with these findings, crotonylation was preferred on lysine residues that adjacent to alanine, glutamate and lysine (Fig. 3c).

### The crotonylated proteins in tea leaves after NH_4_^+^ resupply

From these, we quantified 2164 crotonylation sites on 945 proteins, and then normalized the quantitative lysine crotonylome with the data of the global proteome (Additional file 10). Compared with the NH_4_^+^ deficiency, 232 crotonylation sites on 183 proteins were up-regulated and 72 crotonylation sites on 63 proteins were down-regulated at 3hN based on a fold-change threshold > 1.5, whereas 218 crotonylation sites on 172 proteins were up-regulated and 57 crotonylation sites on 52 proteins were down-regulated at 3dN (Additional file 11). Furthermore, 39 crotonylation sites on 33 proteins were up-regulated and 71 crotonylation sites on 50 proteins were down-regulated at 3dN/3hN. These results proved that lysine crotonylation in tea leaves could directly respond to NH_4_^+^ resupply.

### Design of Venn diagram and functional analysis of lysine crotonylated proteins

To deeply investigate the cellular processes regulated by crotonylation in tea leaves at 3 h and 3d of NH_4_^+^ resupply, a Venn diagram of differentially expressed crotonylated proteins (DCPs) was structured (Additional file 4: Figure S4, Additional file 12). We found that 121 DCPs were specifically observed at 3hN/DN, and 143 DCPs were specifically observed at 3dN/DN. Moreover, 94 DCPs were identical in two conditions. These results proved that the expression of crotonylated proteins was specific at different time points of NH_4_^+^ resupply.

**Fig. 4:**
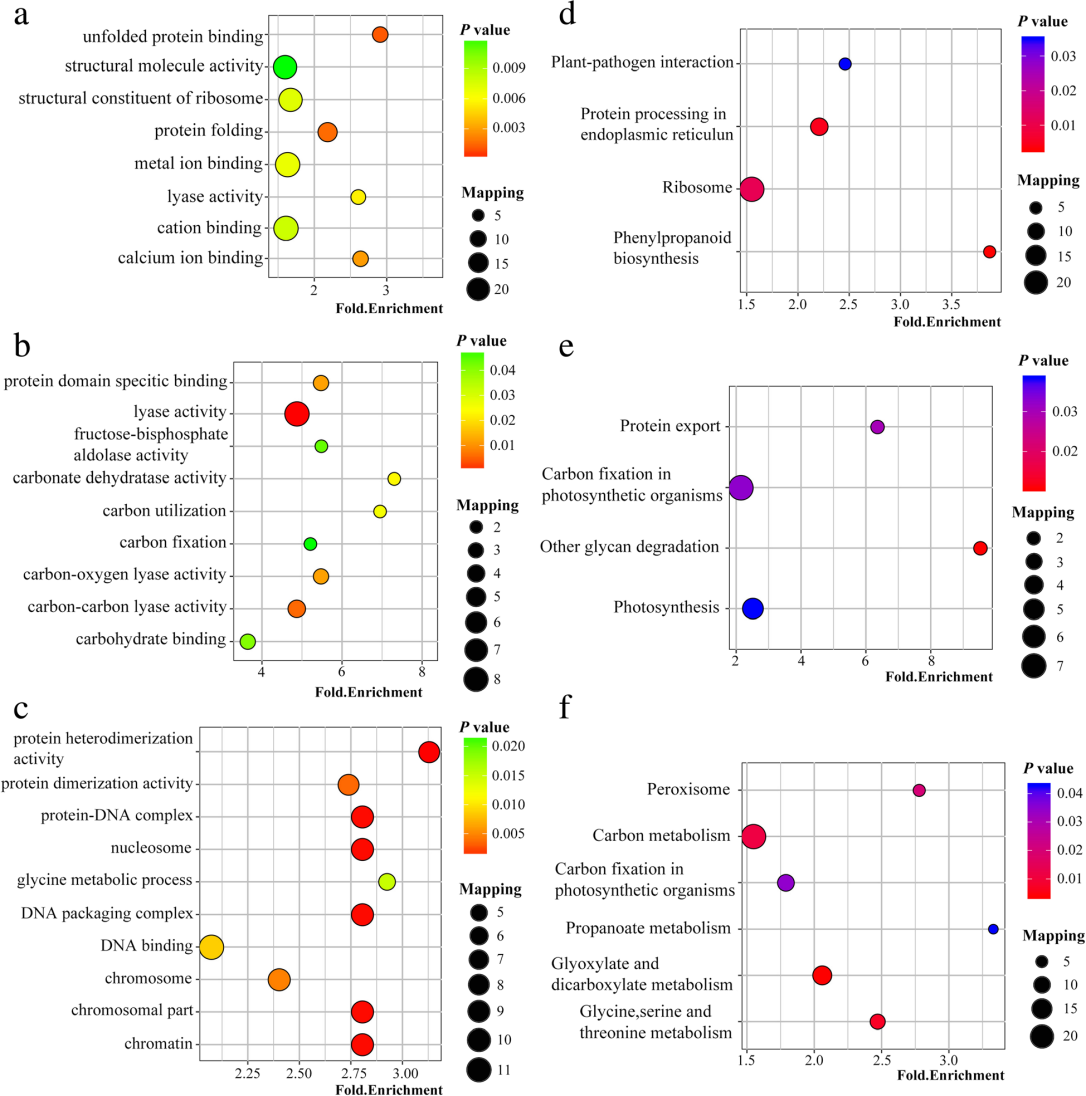
Enrichment analysis of DCPs in tea leaves after NH_4_^+^ resupply. **a** GO enrichment analysis of specific DCPs at 3hN/DN. **b** GO enrichment analysis of common DCPs at 3hN/DN and3dN/DN. **c** GO enrichment analysis of specific DCPs at 3dN/DN. **d** KEGG pathway enrichment analysis of specific DCPs at 3hN/DN. **e** KEGG pathway enrichment analysis of common DCPs at 3hN/DN and3dN/DN. **f** KEGG pathway enrichment analysis of specific DCPs at 3dN/DN

To explore the relevant biological functions and pathways, we performed GO and KEGG enrichment analysis based on the data of Venn analysis (Additional files 13 to 14). The results showed that GO terms of DCPs were enriched exclusively at 3hN/DN, such as structural molecule activity, metal ion binding, cation binding, protein folding and structural constituent of ribosome (Fig. 4a). The GO terms of common DCPs in 3hN/DN and 3dN/DN were similar and both included terms associated with lyase activity, carbon-carbon lyase activity, fructose bisphosphate aldolase activity, carbohydrate binding and carbon fixation (Fig. 4b). Moreover, GO terms of DCPs were enriched exclusively at 3dN/DN, such as protein heterodimerization activity, nucleosome, DNA packaging complex, chromatin and glycine metabolic process (Fig. 4c). These results implied that crotonylated proteins in tea leaves plays an important role in these processes by sensing NH_4_^+^ nutrition.

KEGG pathway analysis showed that 3hN/DN-specific DCPs were enriched to ribosome, protein processing in endoplasmic reticulum, phenylpropanoid biosynthesis and plant−pathogen interaction (Fig. 4d). Furthermore, common DCPs modulated at 3hN/DN and 3dN/DN were enriched to similar pathways, including photosynthesis, carbon fixation in photosynthetic organisms, protein export and other glycan degradation (Fig. 4e). In addition, many 3dN/DN-specific DCPs were enriched to carbon metabolism, glyoxylate and dicarboxylate metabolism, peroxisome, glycine, serine and threonine metabolism (Fig. 4f). In total, crotonylated proteins in tea leaves after NH_4_^+^ resupply was involved in photosynthesis, carbon metabolism, glyoxylate and dicarboxylate metabolism, ribosome, glycine, serine and threonine metabolism.

### The analysis of interaction network in lysine crotonylated proteins

**Fig. 5:**
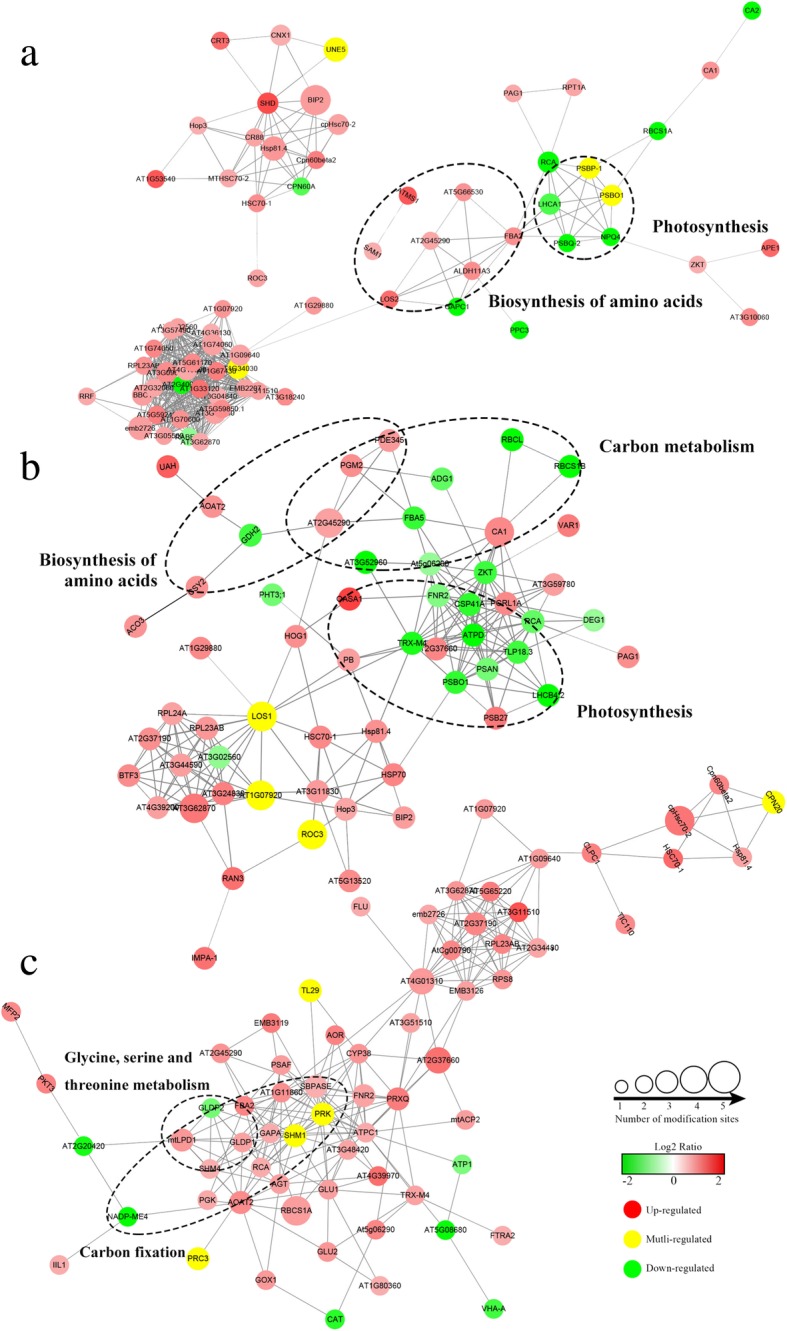
PPI network of DCPs after NH_4_^+^ resupply. **a** The PPI of specific DCPs at 3hN/DN. **b** The PPI of common DCPs at 3hN/DN and3dN/DN, **c** The PPI of specific DCPs at 3dN/DN

To deeply understand the interactions of crotonylated proteins, we constructed the PPI networks for DCPs (Additional file 15). The results showed that 86 specific DCPs were closely connected at 3hN/DN, which mapped to the protein interaction database (Fig. 5a). There was a strong interaction between crotonylated proteins involved in photosynthesis or biosynthesis of amino acids. In this network, 28 crotonylated proteins were identified with the node degree over 10, such as endoplasmin homolog and ALDO. Furthermore, 66 common DCPs were closely connected between 3hN/DN and 3dN/DN (Fig. 5b). There was a close interaction between crotonylated proteins involved in photosynthesis, carbon metabolism and biosynthesis of amino acids. Of which 16 crotonylated proteins were identified with the node degree over 10, such as FNR, PsbO and PsaN. In addition, 70 specific DCPs were closely connected at 3dN/DN (Fig. 5c). There was a close interaction between crotonylated proteins involved in carbon fixation or glycine, serine and threonine metabolism. Thereinto, 21 crotonylated proteins were identified with the node degree over 10, such as SBPase, SHMT, γ-ATPase and PRK. These results can be clearly seen that crotonylated proteins had a close interaction in many primary metabolic processes of tea leaves under NH_4_^+^ deficiency/resupply.

### The changes of crotonylation sites on crotonylated proteins involved in photosynthesis

**Fig. 6:**
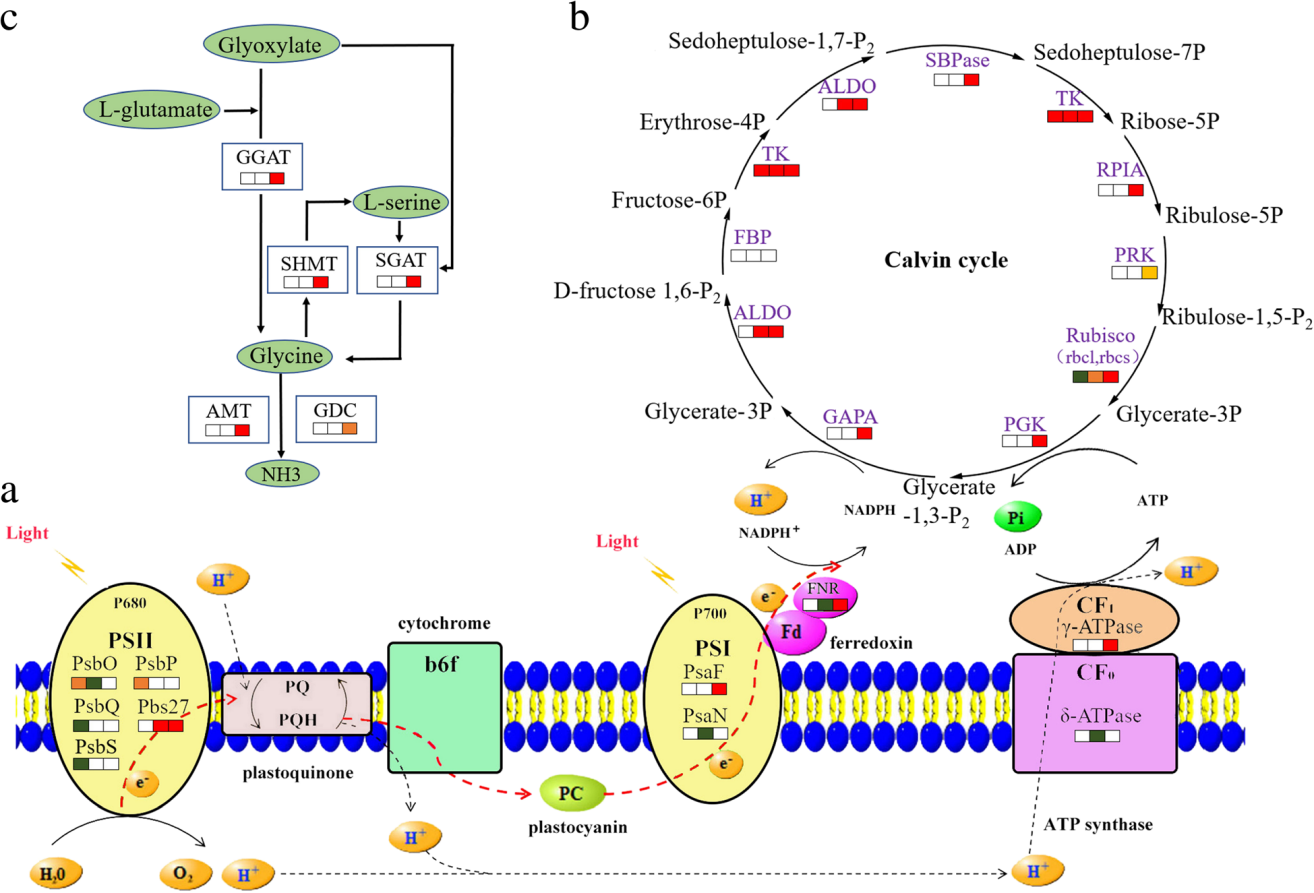
DEPs in involved in primary metabolic processes after NH_4_^+^ resupply. **a** photosynthesis. **b** Calvin cycle. **c** glycine, serine and threonine metabolism. The rectangle was divided into three equal parts (the left of rectangle represented specific DCPs at 3hN/DN; the middle of rectangle represented common DCPs at 3hN/DN and 3dN/DN; the right of rectangle represented specific DCPs at 3dN/DN). The color in the rectangle represents the crotonylated proteins were regulated after NH_4_^+^ resupply (red indicated up-regulation; yellow indicates mutli-regulation; green indicated down-regulation)

To deeply investigate the photosynthesis regulated by lysine crotonylation, we summarized the crotonylated proteins in tea leaves under NH_4_^+^ deficiency/resupply (Fig. 6a, Additional file 16). We found that there were 6 crotonylation sites on 4 specific crotonylated proteins changed obviously at 3hN/DN, containing K270 (up-regulated) and K144 (down-regulated) on PsbO, K119 (down-regulated) and k247 (up-regulated) on PsbP, K147 (down-regulated) on PsbQ and K185 (down-regulated) on PsbS. Meanwhile, there were 5 crotonylation sites on 5 common crotonylated proteins changed significantly between 3hN/DN and 3dN/DN, containing K159 (down-regulated) on PsaN, K121 (down-regulated) on PsbO, K121 (up-regulated) on Pbs27, K238 (down-regulated) on FNR and K117 (down-regulated) on δ-ATPase. Among them, K121 on PsbO and K117 on δ-ATPase decreased more than 3 times and 2 time from 3hN to 3dN, respectively. Furthermore, at 3dN/DN, there were 6 crotonylation sites on 4 specific crotonylated proteins changed significantly, including K124 (up-regulated) on PsaF, K116 (up-regulated) and K169 (up-regulated) on Pbs27, K81 (up-regulated) and K203 (up-regulated) on FNR and K194 (up-regulated) on γ-ATPase.

### The changes of crotonylation sites on crotonylated proteins involved in carbon fixation

To check into the carbon fixation in photosynthetic organisms regulated by lysine crotonylation, we mainly analyzed the crotonylated proteins of Calvin cycle in tea leaves under NH_4_^+^ deficiency/resupply (Fig. 6b, Additional file 17). We found that there were 2 crotonylation sites on 2 specific crotonylated proteins marked change at 3hN/DN, containing K138 (down-regulated) on rbcs and K314 (up-regulated) on TK. Meanwhile, there were 7 crotonylation sites on 4 common crotonylated proteins changed significantly between 3hN/DN and 3dN/DN, such as K190 (up-regulated) on rbcl, K84 (down-regulated) on rbcs, K172 (up-regulated) and K4 (up-regulated) on ALDO, K133 (up-regulated) and K710 (up-regulated) on TK. Furthermore, at 3dN/DN, there were 15 crotonylation sites on 9 specific crotonylated proteins changed highly, including K63 (up-regulated), K110 (up-regulated), K144 (up-regulated) and K160 (up-regulated) on rbcs, K480 (up-regulated) on TK, K207 (up-regulated) and K233 (down-regulated) on PRK, K125 (up-regulated) on PGK, K212 (up-regulated) on RPIA, K80 (up-regulated) on ALDO, K108 (up-regulated) and K305 (up-regulated) on SBPase. Among them, K207 on PRK, K212 on RPIA, K63 and K144 on rbcs were up-regulated from 3 h to 3d of NH_4_^+^ resupply.

### The changes of crotonylation sites on crotonylated proteins involved in amino acid metabolism

To deeply research the amino acid metabolism regulated by lysine crotonylation, especially glycine, serine and threonine (Fig. 6c, Additional file 18), we characterized the crotonylated proteins in tea leaves under NH_4_^+^ deficiency/resupply. Interestingly, there was no significant change in crotonylated proteins at 3hN/DN. However, there were 12 crotonylation sites on 7 specific crotonylated proteins changed dramatically at 3dN/DN, such as K52 (up-regulated) on SGAT, K441 (up-regulated) on SHMT4, K4 (up-regulated), K35 (up-regulated) and K357 (up-regulated) on GGAT2, K478 (up-regulated) and K265 (down-regulated) on GDC, K146 (up-regulated) and (up-regulated) K201 on LPD1. And K146 on LPD1 also was up-regulated from 3hN to 3dN.

### The changes of enzymatic activity and protein contents of DCPs

To investigate whether the activities of DCPs were changed after NH_4_^+^ resupply, we measured the activity of six DCPs involved in Calvin cycle, serine and glycine metabolism, respectively (Additional file 19). Among them, Rubisco (rbcl and rbcs were subunits of Rubisco), TK and PRK were the key enzymes for CO_2_ fixation, reduction of 3-phosphoglycerate and ribulose-1,5-bisphosphate (RUBP) regeneration in Calvin cycle, respectively. Meanwhile, SGAT, GGAT and SHMT were directly involved in serine and glycine synthesis. As a result, the activity of Rubisco, TK and PRK were significantly enhanced after NH_4_^+^ resupply. And the activity of SGAT, GGAT and SHMT were specifically increased at 3dN. Besides, we found that the expression levels of some proteins had no significant change (*F* > 1.5, *P* < 0.05) after NH_4_^+^ resupply which were dramatically expressed at crotonylation level involved in Calvin cycle and serine and glycine synthesis (Additional file 20).

## Discussion

As a kind of evergreen crop, tea plants highly prefer to NH_4_^+^. However, the lysine crotonylation of proteins in tea leaves responding to NH_4_^+^ nutrition has not been studied. Therefore, we investigated the global profiling of lysine crotonylation in tea leaves under NH_4_^+^ deficiency/resupply. A total of 2288 high-confident crotonylation sites on 971 proteins were identified, which greatly expanded the catalog of crotonylated proteins in plants. With the quantitative lysine crotonylome data normalized to the data of the global proteome, we noticed that hundreds of lysine residues and crotonylated proteins changed significantly during NH_4_^+^ resupply. These DCPs were associated with primary metabolism, including photosynthesis, carbon fixation and amino acid metabolism. The PPI network analysis also indicated that a wide range of protein interactions involved in these biological processes was likely modulated by crotonylation.

### DCPs participated in photosynthesis after NH_4_^+^ resupply

Photosynthesis could convert light energy into chemical energy to synthesize NADPH and ATP. Previous research showed that total photosynthetic performances were positively correlated with N content [17]. N deficient leaves damaged capacity for electron transport, thus limiting ATP synthesis and RuBP regeneration [18, 19]. In our study, we noticed that *NC* and *Fv/Fm* showed upward trends after NH_4_^+^ resupply. Moreover, large proportions of crotonylated proteins related to photosynthesis were significantly changed after NH_4_^+^ resupply (Fig. 6a), mainly including PsbO, PsbP, PsbQ, Pbs27, PsaN, PsaF, FNR, γ-ATPase and δ-ATPase, suggesting that lysine crotonylation could play active roles in response to NH_4_^+^ resupply.

PS II was a protein complexes that converted light energy into the electrochemical potential energy required to split water into H^+^, electrons, and molecular oxygen, which was dynamic and constantly underwent assembly, disassembly and repair [20, 21]. However, the rate of repairment and destruction of PS II was unbalanced under various stress conditions, like N deficiency [22, 23]. PsbO, PsbP and PsbQ were extrinsic proteins of PS II complexes, which were indispensable for photosynthetic oxygen evolution and essential for the regulation and stabilization of PS II in higher plants [24, 25]. However, the information about PsbO, PsbP and PsbQ in tea plants were very limited. In this research, the crotonylated PsbO (K121, K144 and K270), PsbP (K119 and K247) and PsbQ (K147) were significantly changed at crotonylation level after NH_4_^+^ resupply. So, our results could provide references for exploring the functions of related proteins and improving the stability of PS II in tea leaves after NH_4_^+^ resupply. In addition, lipoprotein Pbs27 was involved in assembly of the water-splitting site of PS II and the turnover of the complex [26]. In our research, crotonylated Pbs27 was significantly up-regulated at K121 and K116, K121 and K169 at 3hN/DN and 3dN/DN, respectively. This suggested that the crotonylated Psb27 might play a positive function in repair of PS II after NH_4_^+^ resupply. Thus, considering the dynamic changes of crotonylated proteins and the upward trends of *Fv/Fm* after NH_4_^+^ resupply, we speculate that NH_4_^+^ could influence the crotonylation levels of proteins contained in PS II, thus modulating the activity of PS II.

As a sunlight energy converter, the regulation of PS I cyclic electron transport has been considered necessary for photosynthesis and plant growth. N deficiency preferentially affected the activity of PS I, which displayed lower efficiencies for electron flow from intermediate carriers to final PS I acceptors [26]. In PS I, PsaN and PsaF were essential intermediates for electron transferring from plastocyanin to the oxidized reaction centre of P700+. In the present study, crotonylated PsaN (K159) was significantly down-regulated at 3hN/DN and 3dN/DN, while crotonylated PsaF (K124) up-regulated only at 3dN/DN. In addition, we found that crotonylated FNR (K81 and K203), γ-ATPase synthase (K195) and δ-ATPase (K117) were notably changed at 3dN/DN. And K117 on δ-ATPase decreased more than 2 time from 3hN to 3dN. It was reported that FNR had been implicated in cyclic electron transfer around PS I, which catalyzed the last of the light reactions by transferring electrons from ferredoxin to NADP^+^, and was essential for efficient photosynthetic performance [17, 27]. As subunits of ATP synthase, γ-ATPase and δ-ATPase were participated in creating the energy storage molecule ATP, which could facilitate electron transport in both PS I and PS II [28, 29]. From the above, we speculate that lysine crotonylation played important roles in electron transport of PS I in tea leaves after NH_4_^+^ resupply, and specific expression of crotonylated FNR, γ-ATPase and δ-ATPase could have a significant effect on promoting photosynthetic electron transport.

### DCPs involved in the carbon fixation after NH_4_^+^ resupply

Under abiotic stress, particularly in nitrogen nutrition changes, plants gradually adjust the mechanisms of carbon metabolism. Several papers described that N deficiency exacerbated the limitations of photosynthesis performance by reducing the capacity of key enzymes involved in carbon fixation [30, 31]. The Calvin cycle is the primary pathway of carbon fixation. Previous study showed that the transcription levels of genes in the Calvin cycle had distinctly changed when N deficiency seedlings received N [32]. In this work, the almost all enzymes in the Calvin cycle were specifically expressed at crotonylation level after NH_4_^+^ resupply (Fig. 6b), such as rbcs, rbcl, TK, ALDO, PGK and PRK, suggesting that lysine crotonylation participated in the regulation of carbon fixation in tea leaves under NH_4_^+^ resupply.

Rubisco catalyzes the carboxylation of RuBP, which is the first step of the Calvin cycle. Previous studies showed that the content of Rubisco was strictly controlled to maintain C/N balance, and the N source provided by Rubisco degradation under N deficiency [30]. N deficiency reduced the activity of Rubisco in *Arabidopsis* and *Kentucky Bluegrass*, while the activity of Rubisco increased with increasing N content [33, 34]. The results from the current paper indicated that the activity of Rubisco increased after NH_4_^+^ resupply, crotonylated rbcs (K63, K110, K144 and K160) and rbcl (K190) changed significantly. However, the expressions of rbcs and rbcl showed no significant changes at protein level after NH_4_^+^ resupply. So, we speculate that lysine crotonylation was closely related to the activity of Rubisco, and crotonylated Rubisco had a role in regulating the carboxylation of RuBP and the balance of C/N after NH_4_^+^ resupply.

TK centrally locates in the Calvin cycle where it catalyzes the generation of ribose 5-phosphate and erythrose 4-phosphate. Up to now, the crotonylated TK has not been reported in plants. In this research, the activity of TK and the abundance of crotonylated TK were increased after NH_4_^+^ resupply. Because TK was important to maintaining RuBP regeneration and aromatic amino acids synthesis [35, 36]**,** crotonylated TK might be contributing to the regulation of these processes in tea leaves after NH_4_^+^ resupply.

PRK catalyzes the phosphorylation of D-Ribulose 5-phosphate into RuBP, which is the last step of the Calvin cycle. The activity of PRK often determined the metabolic rate in organisms for which carbon fixation was key to survival [37]. Low N reduced the activity and content of PRK in transgenic tobacco, thus limiting photosynthesis and biomass production [38]. In this study, K207 on PRK was increased after NH_4_^+^ resupply. The crotonylated PRK (K207 and K233) was significantly changed at 3dN, and the activity of PRK was increased under NH_4_^+^ deficiency/resupply. As a consequence, we deduce that the crotonylated PRK played key roles in RuBP regeneration after NH_4_^+^ resupply.

### DCPs involved in the amino acid metabolism after NH_4_^+^ resupply

The high levels of amino acids principally contributed to mellowness and freshness in tea [39]. NH_4_^+^ supply was found to effectively enhance the biosynthesis of catechins and free amino acids in tea leaves [1]. Our present study showed that the content of free amino acids was significantly increased after NH_4_^+^ resupply, and large amounts of enzymes participated in glycine, serine and threonine metabolism were specifically changed at crotonylation level after 3dN, not at 3hN (Fig. 6c), suggesting that lysine crotonylation could selectively participate in the amino acid metabolism under NH_4_^+^ deficiency/resupply.

GGAT and SGAT can function cooperatively in the production of glycine. Previous research showed that the activity of GGAT was sharply down-regulated and the activity of SGAT was little affected in rice under N deficiency. The substrate glutamate was accumulated while downstream products of glycine and serine were significantly reduced, indicated that the glycine formation from glyoxylate relies sensitively on GGAT than SGAT [40]. However, the response mechanism of SGAT and GGAT at crotonylation level have not been reported. In this research, we found that the activity of GGAT and SGAT were increased at 3dN, and crotonylated GGAT (K4, K35 and K367) and SGAT (K52) were significantly up-regulated, while GGAT and SGAT were not significantly changed at protein level after NH_4_^+^ resupply. We contemplate that lysine crotonylation might participate in regulating the activity of GGAT and SGAT after NH_4_^+^ resupply, but it still needs further investigation. In addition, according to previous reports, the improvement of GGAT activity could enhance the efficiency of nitrate and ammonium assimilation [41]. Thus, the crotonylated GGAT might plays important roles in N metabolism and glycine formation in tea leaves after NH_4_^+^ resupply.

SHMT is an essential player in the serine homeostasis, which catalyzes the reversible serine to glycine conversion. In microalgae, the SHMT might aid in maintaining cellular homeostasis during later stages of N deficiency [42]. In *Agrostis stolonifera*, the abundance of SHMT decreased with N deficiency, affecting the biosynthesis of amino acids and lipids [43]. In this study, the abundance of SHMT showed no remarkable change under NH_4_^+^ deficiency/resupply, while the activity of SHMT and the abundance of crotonylated SHMT4 (SHMT subunit) were significantly increased at 3dN/DN. We have reason to deduce that the crotonylated SHMT4 might affect the activity of SHMT and then consequently regulate the amino acids homeostasis in tea leaves after NH_4_^+^ resupply.

## Conclusion

Above all, lysine crotonylation in tea leaves subjected to NH_4_^+^ deficiency/resupply was described as a pioneering study of crop. We quantified 2288 crotonylation sites on 971 proteins in tea leaves. After NH_4_^+^resupply, the crotonylated proteins played key roles in primary metabolic processes, especially in photosynthesis, carbon fixation and amino acid metabolism in tea leaves. Furthermore, the results of analysis implied that lysine crotonylation was associated with activities of certain enzymes by sensing NH_4_^+^. The ultimate function of the crotonylated proteins requires further thoroughly research by site-specific crotonylation antibodies. The information obtained from the lysine crotonylation could be helpful in exploring metabolic mechanism of proteins of tea plants in response to N.

## Additional files

Additional file 1: The nutrient solution formula. (XLSX 10 kb)

Additional file 2: The detailed description of protein extraction and trypsin digestion. (DOCX 18 kb)

Additional file 3: The methods of enzyme activity determination. (DOCX 49 kb)

Additional file 4: **Figure S1.** The physiological analyses of tea leaves after NH_4_^+^ resupply. **Figure S2.** SDS-PAGE of three samples under NH_4_^+^ deficiency/resupply. **Figure S3.** The number of crotonylation sites identified per protein. **Figure S4.** The Venn diagram analysis of DCPs at 3 h and 3d of NH_4_^+^ resupply. (DOCX 343 kb)

Additional file 5: The information of lysine crotonylome. (XLSX 3355 kb)

Additional file 6: Fifty representative LC-MS/MS spectra of crotonylated peptides. (DOCX 8024 kb)

Additional file 7: The information of global proteome. (XLSX 1932 kb)

Additional file 8: The information of subcellular localization. (XLSX 326 kb)

Additional file 9: The information of motif-X. (XLSX 505 kb)

Additional file 10: The data of normalization. (XLSX 717 kb)

Additional file 11: The information of DCPs. (XLSX 219 kb)

Additional file 12: The information of Venn diagram. (XLSX 76 kb)

Additional file 13: The information of GO enrichment analysis of DCPs. (XLSX 18 kb)

Additional file 14: The information of KEGG enrichment analysis of DCPs. (XLSX 14 kb)

Additional file 15: The information of PPI analysis of DCPs. (XLSX 45 kb)

Additional file 16: The information of DCPs involved in photosynthesis. (XLSX 12 kb)

Additional file 17: The information of DCPs involved in carbon fixation. (XLSX 14 kb)

Additional file 18: The information of DCPs involved in amino acid metabolism. (XLSX 11 kb)

Additional file 19: The enzyme activities of DCPs after NH_4_^+^ resupply. (DOCX 183 kb)

Additional file 20: The information of proteins was concerned with DCPs. (XLSX 11 kb)

## Abbreviations

ALDO: Fructose-bisphosphate aldolase
FNR: Ferredoxin--NADP reductase
GDC: Glycine dehydrogenase
GGAT2: Glutamate-glyoxylate aminotransferase 2-like
LPD1: Dihydrolipoyl dehydrogenase 1
Pbs27: Photosystem II Pbs27 protein
PGK: Phosphoglycerate kinase
PRK: Phosphoribulokinase
PsaF: Photosystem I reaction center subunit III
PsaN: Photosystem I reaction center subunit N
PsbO: Oxygen-evolving enhancer protein 1
PsbP: Oxygen-evolving enhancer protein 2
PsbQ: Oxygen-evolving enhancer protein 3–2
PsbS: Photosystem II 22 kDa protein
rbcl: Ribulose bisphosphate carboxylase large chain
rbcs: Ribulose bisphosphate carboxylase small chain
RPIA: Ribose-5-phosphate isomerase 3
Rubisco: Ribulose bisphosphate carboxylase oxygenase
RuBP: Ribulose-1,5-bisphosphate
SBPase: Sedoheptulose-1,7-bisphosphatase
SGAT: Serine-glyoxylate aminotransferase
SHMT4: Serine hydroxymethyltransferase 4
TK: Transketolase
γ-ATPase: ATP synthase gamma chain
δ-ATPase: ATP synthase delta chain
