# Supplementary material for: Ammonium triggered the response mechanism of lysine crotonylome in tea plants
Source: BMC Genomics. 2019 May 6;20:340. doi: 10.1186/s12864-019-5716-z (PMC6501322; doi:10.1186/s12864-019-5716-z)
Supplement: Supplementary file 2 — The detailed description of protein extraction and trypsin digestion. (DOCX 18 kb) [file 12864_2019_5716_MOESM2_ESM.docx]

**Protein Extraction**

The sample was grinded by liquid nitrogen into cell powder and then transferred to a 5-mL centrifuge tube. After that, four volumes of lysis buffer (8 M urea, 1% Triton-100, 10 mM dithiothreitol, and 1% Protease Inhibitor Cocktail) was added to the cell powder, followed by sonication three times on ice using a high intensity ultrasonic processor (Scientz). (Note: For PTM experiments, inhibitors were also added to the lysis buffer, e.g. 3 μM TSA and 50 mM NAM for acetylation.) The remaining debris was removed by centrifugation at 20,000 at 4 °C for 10 min. Finally, the protein was precipitated with cold 20% TCA for 2 hours at -20 °C. After centrifugation at 12,000 g 4 °C for 10 min, the supernatant was discarded. The remaining precipitate was washed with cold acetone for three times. The protein was redissolved in 8 M urea and the protein concentration was determined with BCA kit according to the manufacturer’s instructions.

**Trypsin Digestion**

For digestion, the protein solution was reduced with 5 mM dithiothreitol for 30 min at 56 °C and alkylated with 11 mM iodoacetamide for 15 min at room temperature in darkness. The protein sample was then diluted by adding 100 mM NH_4_HCO_3_ to urea concentration less than 2M. Finally, trypsin was added at 1:50 trypsin-to-protein mass ratio for the first digestion overnight and 1:100 trypsin-to-protein mass ratio for a second 4 h-digestion.
