# Supplementary material for: Ammonium triggered the response mechanism of lysine crotonylome in tea plants
Source: BMC Genomics. 2019 May 6;20:340. doi: 10.1186/s12864-019-5716-z (PMC6501322; doi:10.1186/s12864-019-5716-z)
Supplement: Supplementary file 3 — The methods of enzyme activity determination. (DOCX 49 kb) [file 12864_2019_5716_MOESM3_ESM.docx]

**Rubisco**

**Principle of the assay**

The kit assay Plant RUBISCO level in the sample，use Purified Plant RUBISCO antibody to coat microtiter plate wells, make solid-phase antibody, then add RUBISCO to wells, Combined RUBISCO antibody which With HRP labeled, become antibody - antigen - enzyme-antibody complex, after washing Completely, Add TMB substrate solution, TMB substrate becomes blue color At HRP enzyme-catalyzed, reaction is terminated by the addition of a sulphuric acid solution and the color change is measured spectrophotometrically at a wavelength of 450 nm. The concentration of Plant RUBISCO in the samples is then determined by comparing the O.D. of the samples to the standard curve.

**Materials provided with the k**it

| 1 | wash solution | 20ml×1bottle | 7 | Stop Solution | 6ml×1 bottle |
| --- | --- | --- | --- | --- | --- |
| 2 | HRP-Conjugate reagent | 6ml×1 bottle | 8 | Standard（1200U/L） | 0.5ml×1 bottle |
| 3 | Microelisa stripplate | 12well×8strips | 9 | Standard diluent | 1.5ml×1bottle |
| 4 | Sample diluent | 6ml×1 bottle | 10 | Instruction | 1 |
| 5 | Chromogen Solution A | 6ml×1 bottle | 11 | Closure plate membrane | 2 |
| 6 | Chromogen Solution B | 6ml×1 bottle | 12 | Sealed bags | 1 |

**Specimen requirements**

1. extract as soon as possible after Specimen collection, and according to the relevant literature, and should be experiment as soon as possible after the extraction. If it can’t, specimen can be kept in -20 ℃ to preserve, Avoid repeated freeze-thaw cycles.
2. Can’t detect the sample which contain NaN3, because NaN3 inhibits HRP active.

**Assay procedure**

1. Dilute and add sample: Dilute Original density Standard as follow table:

| 600U/L | 5 Standard | 150μl Original density Standard+150μl Standard diluent |
| --- | --- | --- |
| 300U/L | 4 Standard | 150μl 5 Standard+150μl Standard diluent |
| 150U/L | 3 Standard | 150μl 4 Standard+150μl Standard diluent |
| 75U/L | 2 Standard | 150μl 3 Standard +150μl Standard diluent |
| 37.5 U/L | 1 Standard | 150μl 2 Standard +150μl Standard diluent |

2.add sample：Set blank wells separately (blank comparison wells don’t add sample and HRP-Conjugate reagent, other each step operation is same). testing sample well. add Sample dilution 40μl to testing sample well, then add testing sample 10μl (sample final dilution is 5-fold), add sample to wells, don’t touch the well wall as far as possible, and Gently mix.

3.Incubate: After closing plate with Closure plate membrane, incubate for 30 min at 37℃.

4.Configurate liquid: 30-fold (or 20-fold) wash solution diluted 30-fold (or 20-fold) with distilled water and reserve.

5.washing：Uncover Closure plate membrane, discard Liquid, dry by swing, add washing buffer to every well, still for 30s then drain, repeat 5 times, dry by pat.

6.add enzyme：Add HRP-Conjugate reagent 50μl to each well, except blank well.

7.incubate：Operation with 3.

8.washing：Operation with 5.

9.color：Add Chromogen Solution A 50ul and Chromogen Solution B 50ul to each well, evade the light preservation for 10 min at 37℃

10.Stop the reaction：Add Stop Solution50μl to each well, Stop the reaction (the blue color change to yellow color).

11.assay：Take blank well as zero, Read absorbance at 450nm after Adding Stop Solution and within 15min.

**Calculate**

Take the standard density as the horizontal, the OD value for the vertical ,draw the standard curve on graph paper, Find out the corresponding density according to the sample OD value by the Sample curve, multiplied by the dilution multiple, or calculate the straight line regression equation of the standard curve with the standard density and the OD value ,with the sample OD value in the equation, calculate the sample density, multiplied by the dilution factor, the result is the sample actual density.

**TK**

**Principle of the assay**

The kit assay Plant TK level in the sample，use Purified Plant TK antibody to coat microtiter plate wells, make solid-phase antibody, then add TK to wells, Combined TK antibody which With HRP labeled, become antibody - antigen - enzyme-antibody complex, after washing Completely, Add TMB substrate solution, TMB substrate becomes blue color At HRP enzyme-TKalyzed, reaction is terminated by the addition of a sulphuric acid solution and the color change is measured spectrophotometrically at a wavelength of 450 nm. The concentration of Plant TK in the samples is then determined by comparing the O.D. of the samples to the standard curve.

**Materials provided with the k**it

| 1 | wash solution | 20ml×1bottle | 7 | Stop Solution | 6ml×1 bottle |
| --- | --- | --- | --- | --- | --- |
| 2 | HRP-Conjugate reagent | 6ml×1 bottle | 8 | Standard（1200U/L） | 0.5ml×1 bottle |
| 3 | Microelisa stripplate | 12well×8strips | 9 | Standard diluent | 1.5ml×1bottle |
| 4 | Sample diluent | 6ml×1 bottle | 10 | Instruction | 1 |
| 5 | Chromogen Solution A | 6ml×1 bottle | 11 | Closure plate membrane | 2 |
| 6 | Chromogen Solution B | 6ml×1 bottle | 12 | Sealed bags | 1 |

**Specimen requirements**

1. extract as soon as possible after Specimen collection, and according to the relevant literature, and should be experiment as soon as possible after the extraction. If it can’t, specimen can be kept in -20 ℃ to preserve, Avoid repeated freeze-thaw cycles.
2. Can’t detect the sample which contain NaN3, because NaN3 inhibits HRP active.

**Assay procedure**

1. Dilute and add sample: Dilute Original density Standard as follow table:

| 600U/L | 5 Standard | 150μl Original density Standard+150μl Standard diluent |
| --- | --- | --- |
| 300U/L | 4 Standard | 150μl 5 Standard+150μl Standard diluent |
| 150U/L | 3 Standard | 150μl 4 Standard+150μl Standard diluent |
| 75U/L | 2 Standard | 150μl 3 Standard +150μl Standard diluent |
| 37.5 U/L | 1 Standard | 150μl 2 Standard +150μl Standard diluent |

2.add sample：Set blank wells separately (blank comparison wells don’t add sample and HRP-Conjugate reagent, other each step operation is same). testing sample well. add Sample dilution 40μl to testing sample well, then add testing sample 10μl (sample final dilution is 5-fold), add sample to wells, don’t touch the well wall as far as possible, and Gently mix.

3.Incubate: After closing plate with Closure plate membrane, incubate for 30 min at 37℃.

4.Configurate liquid: 30-fold (or 20-fold) wash solution diluted 30-fold (or 20-fold) with distilled water and reserve.

5.washing：Uncover Closure plate membrane, discard Liquid, dry by swing, add washing buffer to every well, still for 30s then drain, repeat 5 times, dry by pat.

6.add enzyme：Add HRP-Conjugate reagent 50μl to each well, except blank well.

7.incubate：Operation with 3.

8.washing：Operation with 5.

9.color：Add Chromogen Solution A 50ul and Chromogen Solution B to each well, evade the light preservation for 10 min at 37℃

10.Stop the reaction：Add Stop Solution50μl to each well, Stop the reaction (the blue color change to yellow color).

11.assay：Take blank well as zero, Read absorbance at 450nm after Adding Stop Solution and within 15min.

**Calculate**

Take the standard density as the horizontal, the OD value for the vertical ,draw the standard curve on graph paper, Find out the corresponding density according to the sample OD value by the Sample curve, multiplied by the dilution multiple, or calculate the straight line regression equation of the standard curve with the standard density and the OD value ,with the sample OD value in the equation, calculate the sample density, multiplied by the dilution factor, the result is the sample actual density.

**GGAT**

**Principle of the assay**

The kit assay Plant GGAT level in the sample，use Purified Plant GGAT antibody to coat microtiter plate wells, make solid-phase antibody, then add GGAT to wells, Combined GGAT antibody which With HRP labeled, become antibody - antigen - enzyme-antibody complex, after washing Completely, Add TMB substrate solution, TMB substrate becomes blue color At HRP enzyme-GGATalyzed, reaction is terminated by the addition of a sulphuric acid solution and the color change is measured spectrophotometrically at a wavelength of 450 nm. The concentration of Plant GGAT in the samples is then determined by comparing the O.D. of the samples to the standard curve.

**Materials provided with the k**it

| 1 | wash solution | 20ml×1bottle | 7 | Stop Solution | 6ml×1 bottle |
| --- | --- | --- | --- | --- | --- |
| 2 | HRP-Conjugate reagent | 6ml×1 bottle | 8 | Standard（800U/L） | 0.5ml×1 bottle |
| 3 | Microelisa stripplate | 12well×8strips | 9 | Standard diluent | 1.5ml×1bottle |
| 4 | Sample diluent | 6ml×1 bottle | 10 | Instruction | 1 |
| 5 | Chromogen Solution A | 6ml×1 bottle | 11 | Closure plate membrane | 2 |
| 6 | Chromogen Solution B | 6ml×1 bottle | 12 | Sealed bags | 1 |

**Specimen requirements**

1. extract as soon as possible after Specimen collection, and according to the relevant literature, and should be experiment as soon as possible after the extraction. If it can’t, specimen can be kept in -20 ℃ to preserve, Avoid repeated freeze-thaw cycles.
2. Can’t detect the sample which contain NaN3, because NaN3 inhibits HRP active.

**Assay procedure**

1. Dilute and add sample: Dilute Original density Standard as follow table:

| 400U/L | 5 Standard | 150μl Original density Standard+150μl Standard diluent |
| --- | --- | --- |
| 200U/L | 4 Standard | 150μl 5 Standard+150μl Standard diluent |
| 100U/L | 3 Standard | 150μl 4 Standard+150μl Standard diluent |
| 50U/L | 2 Standard | 150μl 3 Standard +150μl Standard diluent |
| 25 U/L | 1 Standard | 150μl 2 Standard +150μl Standard diluent |

2.add sample：Set blank wells separately (blank comparison wells don’t add sample and HRP-Conjugate reagent, other each step operation is same). testing sample well. add Sample dilution 40μl to testing sample well, then add testing sample 10μl (sample final dilution is 5-fold), add sample to wells, don’t touch the well wall as far as possible, and Gently mix.

3.Incubate: After closing plate with Closure plate membrane, incubate for 30 min at 37℃.

4.Configurate liquid: 30-fold (or 20-fold) wash solution diluted 30-fold (or 20-fold) with distilled water and reserve.

5.washing：Uncover Closure plate membrane, discard Liquid, dry by swing, add washing buffer to every well, still for 30s then drain, repeat 5 times, dry by pat.

6.add enzyme：Add HRP-Conjugate reagent 50μl to each well, except blank well.

7.incubate：Operation with 3.

8.washing：Operation with 5.

9.color：Add Chromogen Solution A 50ul and Chromogen Solution B to each well, evade the light preservation for 10 min at 37℃

10.Stop the reaction：Add Stop Solution50μl to each well, Stop the reaction (the blue color change to yellow color).

11.assay：Take blank well as zero, Read absorbance at 450nm after Adding Stop Solution and within 15min.

**Calculate**

Take the standard density as the horizontal, the OD value for the vertical ,draw the standard curve on graph paper, Find out the corresponding density according to the sample OD value by the Sample curve, multiplied by the dilution multiple, or calculate the straight line regression equation of the standard curve with the standard density and the OD value ,with the sample OD value in the equation, calculate the sample density, multiplied by the dilution factor, the result is the sample actual density.

**SGAT**

**Principle of the assay**

The kit assay Plant SGAT level in the sample，use Purified Plant SGAT antibody to coat microtiter plate wells, make solid-phase antibody, then add SGAT to wells, Combined SGAT antibody which With HRP labeled, become antibody - antigen - enzyme-antibody complex, after washing Completely, Add TMB substrate solution, TMB substrate becomes blue color At HRP enzyme-catalyzed, reaction is terminated by the addition of a sulphuric acid solution and the color change is measured spectrophotometrically at a wavelength of 450 nm. The concentration of Plant SGAT in the samples is then determined by comparing the O.D. of the samples to the standard curve.

**Materials provided with the k**it

| 1 | wash solution | 20ml×1bottle | 7 | Stop Solution | 6ml×1 bottle |
| --- | --- | --- | --- | --- | --- |
| 2 | HRP-Conjugate reagent | 6ml×1 bottle | 8 | Standard（1200U/L） | 0.5ml×1 bottle |
| 3 | Microelisa stripplate | 12well×8strips | 9 | Standard diluent | 1.5ml×1bottle |
| 4 | Sample diluent | 6ml×1 bottle | 10 | Instruction | 1 |
| 5 | Chromogen Solution A | 6ml×1 bottle | 11 | Closure plate membrane | 2 |
| 6 | Chromogen Solution B | 6ml×1 bottle | 12 | Sealed bags | 1 |

**Specimen requirements**

1. extract as soon as possible after Specimen collection, and according to the relevant literature, and should be experiment as soon as possible after the extraction. If it can’t, specimen can be kept in -20 ℃ to preserve, Avoid repeated freeze-thaw cycles.
2. Can’t detect the sample which contain NaN3, because NaN3 inhibits HRP active.

**Assay procedure**

1. Dilute and add sample: Dilute Original density Standard as follow table:

| 600IU/L | 5 Standard | 150μl Original density Standard+150μl Standard diluent |
| --- | --- | --- |
| 300IU/L | 4 Standard | 150μl 5 Standard+150μl Standard diluent |
| 150IU/L | 3 Standard | 150μl 4 Standard+150μl Standard diluent |
| 75IU/L | 2 Standard | 150μl 3 Standard +150μl Standard diluent |
| 37.5IU/L | 1 Standard | 150μl 2 Standard +150μl Standard diluent |

2.add sample：Set blank wells separately (blank comparison wells don’t add sample and HRP-Conjugate reagent, other each step operation is same). testing sample well. add Sample dilution 40μl to testing sample well, then add testing sample 10μl (sample final dilution is 5-fold), add sample to wells, don’t touch the well wall as far as possible, and Gently mix.

3.Incubate: After closing plate with Closure plate membrane, incubate for 30 min at 37℃.

4.Configurate liquid: 30-fold (or 20-fold) wash solution diluted 30-fold (or 20-fold) with distilled water and reserve.

5.washing：Uncover Closure plate membrane, discard Liquid, dry by swing, add washing buffer to every well, still for 30s then drain, repeat 5 times, dry by pat.

6.add enzyme：Add HRP-Conjugate reagent 50μl to each well, except blank well.

7.incubate：Operation with 3.

8.washing：Operation with 5.

9.color：Add Chromogen Solution A 50ul and Chromogen Solution B 50ul to each well, evade the light preservation for 10 min at 37℃

10.Stop the reaction：Add Stop Solution50μl to each well, Stop the reaction (the blue color change to yellow color).

11.assay：Take blank well as zero, Read absorbance at 450nm after Adding Stop Solution and within 15min.

**Calculate**

Take the standard density as the horizontal, the OD value for the vertical ,draw the standard curve on graph paper, Find out the corresponding density according to the sample OD value by the Sample curve, multiplied by the dilution multiple, or calculate the straight line regression equation of the standard curve with the standard density and the OD value ,with the sample OD value in the equation, calculate the sample density, multiplied by the dilution factor, the result is the sample actual density.

**SHMT**

**Principle of the assay**

The kit assay Plant SHMT level in the sample，use Purified Plant SHMT antibody to coat microtiter plate wells, make solid-phase antibody, then add SHMT to wells, Combined SHMT antibody which With HRP labeled, become antibody - antigen - enzyme-antibody complex, after washing Completely, Add TMB substrate solution, TMB substrate becomes blue color At HRP enzyme-catalyzed, reaction is terminated by the addition of a sulphuric acid solution and the color change is measured spectrophotometrically at a wavelength of 450 nm. The concentration of Plant SHMT in the samples is then determined by comparing the O.D. of the samples to the standard curve.

**Materials provided with the k**it

| 1 | wash solution | 20ml×1bottle | 7 | Stop Solution | 6ml×1 bottle |
| --- | --- | --- | --- | --- | --- |
| 2 | HRP-Conjugate reagent | 6ml×1 bottle | 8 | Standard（800U/L） | 0.5ml×1 bottle |
| 3 | Microelisa stripplate | 12well×8strips | 9 | Standard diluent | 1.5ml×1bottle |
| 4 | Sample diluent | 6ml×1 bottle | 10 | Instruction | 1 |
| 5 | Chromogen Solution A | 6ml×1 bottle | 11 | Closure plate membrane | 2 |
| 6 | Chromogen Solution B | 6ml×1 bottle | 12 | Sealed bags | 1 |

**Specimen requirements**

1. extract as soon as possible after Specimen collection, and according to the relevant literature, and should be experiment as soon as possible after the extraction. If it can’t, specimen can be kept in -20 ℃ to preserve, Avoid repeated freeze-thaw cycles.
2. Can’t detect the sample which contain NaN3, because NaN3 inhibits HRP active.

**Assay procedure**

1. Dilute and add sample: Dilute Original density Standard as follow table:

| 400U/L | 5 Standard | 150μl Original density Standard+150μl Standard diluent |
| --- | --- | --- |
| 200U/L | 4 Standard | 150μl 5 Standard+150μl Standard diluent |
| 100U/L | 3 Standard | 150μl 4 Standard+150μl Standard diluent |
| 50U/L | 2 Standard | 150μl 3 Standard +150μl Standard diluent |
| 25U/L | 1 Standard | 150μl 2 Standard +150μl Standard diluent |

2.add sample：Set blank wells separately (blank comparison wells don’t add sample and HRP-Conjugate reagent, other each step operation is same). testing sample well. add Sample dilution 40μl to testing sample well, then add testing sample 10μl (sample final dilution is 5-fold), add sample to wells, don’t touch the well wall as far as possible, and Gently mix.

3.Incubate: After closing plate with Closure plate membrane, incubate for 30 min at 37℃.

4.Configurate liquid: 30-fold (or 20-fold) wash solution diluted 30-fold (or 20-fold) with distilled water and reserve.

5.washing：Uncover Closure plate membrane, discard Liquid, dry by swing, add washing buffer to every well, still for 30s then drain, repeat 5 times, dry by pat.

6.add enzyme：Add HRP-Conjugate reagent 50μl to each well, except blank well.

7.incubate：Operation with 3.

8.washing：Operation with 5.

9.color：Add Chromogen Solution A 50ul and Chromogen Solution B 50ul to each well, evade the light preservation for 10 min at 37℃

10.Stop the reaction：Add Stop Solution50μl to each well, Stop the reaction (the blue color change to yellow color).

11.assay：Take blank well as zero, Read absorbance at 450nm after Adding Stop Solution and within 15min.

**Calculate**

Take the standard density as the horizontal, the OD value for the vertical ,draw the standard curve on graph paper, Find out the corresponding density according to the sample OD value by the Sample curve, multiplied by the dilution multiple, or calculate the straight line regression equation of the standard curve with the standard density and the OD value ,with the sample OD value in the equation, calculate the sample density, multiplied by the dilution factor, the result is the sample actual density.

**Plant RuPK**

**Principle of the assay**

The kit assay Plant RUPK level in the sample，use Purified Plant RUPK antibody to coat microtiter plate wells, make solid-phase antibody, then add RUPK to wells, Combined RUPK antibody which With HRP labeled, become antibody - antigen - enzyme-antibody complex, after washing Completely, Add TMB substrate solution, TMB substrate becomes blue color At HRP enzyme-catalyzed, reaction is terminated by the addition of a sulphuric acid solution and the color change is measured spectrophotometrically at a wavelength of 450 nm. The concentration of Plant RUPK in the samples is then determined by comparing the O.D. of the samples to the standard curve.

**Materials provided with the kit**

| 1 | wash solution | 20ml×1bottle | 7 | Stop Solution | 6ml×1 bottle |
| --- | --- | --- | --- | --- | --- |
| 2 | HRP-Conjugate reagent | 6ml×1 bottle | 8 | Standard（640U/L） | 0.5ml×1 bottle |
| 3 | Microelisa stripplate | 12well×8strips | 9 | Standard diluent | 1.5ml×1bottle |
| 4 | Sample diluent | 6ml×1 bottle | 10 | Instruction | 1 |
| 5 | Chromogen Solution A | 6ml×1 bottle | 11 | Closure plate membrane | 2 |
| 6 | Chromogen Solution B | 6ml×1 bottle | 12 | Sealed bags | 1 |

**Specimen requirements**

1. extract as soon as possible after Specimen collection, and according to the relevant literature, and should be experiment as soon as possible after the extraction. If it can’t, specimen can be kept in -20 ℃ to preserve, Avoid repeated freeze-thaw cycles.
2. Can’t detect the sample which contain NaN3, because NaN3 inhibits HRP active.

**Assay procedure**

1. Dilute and add sample: Dilute Original density Standard as follow table:

| 320U/L | 5 Standard | 150μl Original density Standard+150μl Standard diluent |
| --- | --- | --- |
| 160U/L | 4 Standard | 150μl 5 Standard+150μl Standard diluent |
| 80U/L | 3 Standard | 150μl 4 Standard+150μl Standard diluent |
| 40U/L | 2 Standard | 150μl 3 Standard +150μl Standard diluent |
| 20U/L | 1 Standard | 150μl 2 Standard +150μl Standard diluent |

2.add sample：Set blank wells separately (blank comparison wells don’t add sample and HRP-Conjugate reagent, other each step operation is same). testing sample well. add Sample dilution 40μl to testing sample well, then add testing sample 10μl (sample final dilution is 5-fold), add sample to wells, don’t touch the well wall as far as possible, and Gently mix.

3.Incubate: After closing plate with Closure plate membrane, incubate for 30 min at 37℃.

4.Configurate liquid: 30-fold (or 20-fold) wash solution diluted 30-fold (or 20-fold) with distilled water and reserve.

5.washing：Uncover Closure plate membrane, discard Liquid, dry by swing, add washing buffer to every well, still for 30s then drain, repeat 5 times, dry by pat.

6.add enzyme：Add HRP-Conjugate reagent 50μl to each well, except blank well.

7.incubate：Operation with 3.

8.washing：Operation with 5.

9.color：Add Chromogen Solution A 50ul and Chromogen Solution B 50ul to each well, evade the light preservation for 10 min at 37℃

10.Stop the reaction：Add Stop Solution50μl to each well, Stop the reaction (the blue color change to yellow color).

11.assay：Take blank well as zero, Read absorbance at 450nm after Adding Stop Solution and within 15min.

**Calculate**

Take the standard density as the horizontal, the OD value for the vertical ,draw the standard curve on graph paper, Find out the corresponding density according to the sample OD value by the Sample curve, multiplied by the dilution multiple, or calculate the straight line regression equation of the standard curve with the standard density and the OD value ,with the sample OD value in the equation, calculate the sample density, multiplied by the dilution factor, the result is the sample actual density.
