# Supplementary material for: Ammonium triggered the response mechanism of lysine crotonylome in tea plants
Source: BMC Genomics. 2019 May 6;20:340. doi: 10.1186/s12864-019-5716-z (PMC6501322; doi:10.1186/s12864-019-5716-z)
Supplement: Supplementary file 4 — Figure S1. The physiological analyses of tea leaves after NH4+ resupply. Figure S2. SDS-PAGE of three samples under NH4+ deficiency/resupply. Figure S3. The number of crotonylation sites identified per protein. Figure S4. The Venn diagram analysis of DCPs at 3 h and 3d of NH4+ resupply. (DOCX 343 kb) [file 12864_2019_5716_MOESM4_ESM.docx]

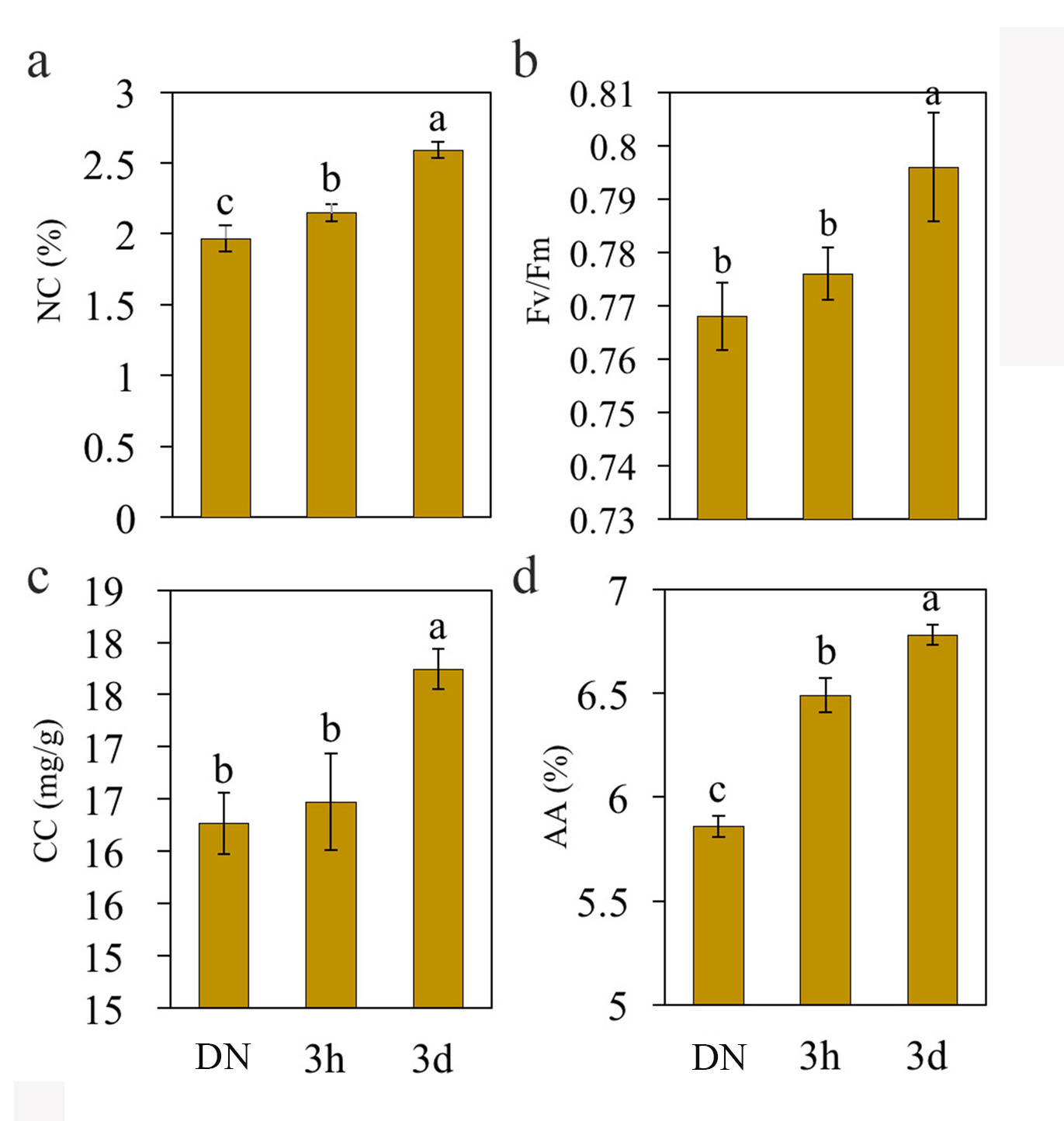


**Fig. S1** The physiological analyses of tea leaves after NH_4_^+^ resupply. **a** Leaf nitrogen content (NC), **b** leaf maximum photochemical quantum yield of PS II (Fv/Fm), **c** leaf chlorphyll content (CC), and **d** Free amino acid content (AA) were determined at three time-points (DN, 3h and 3d) after NH_4_^+^ resupply. Bars (a, b or c) indicate mean values ± standard errors, p < 0.05.


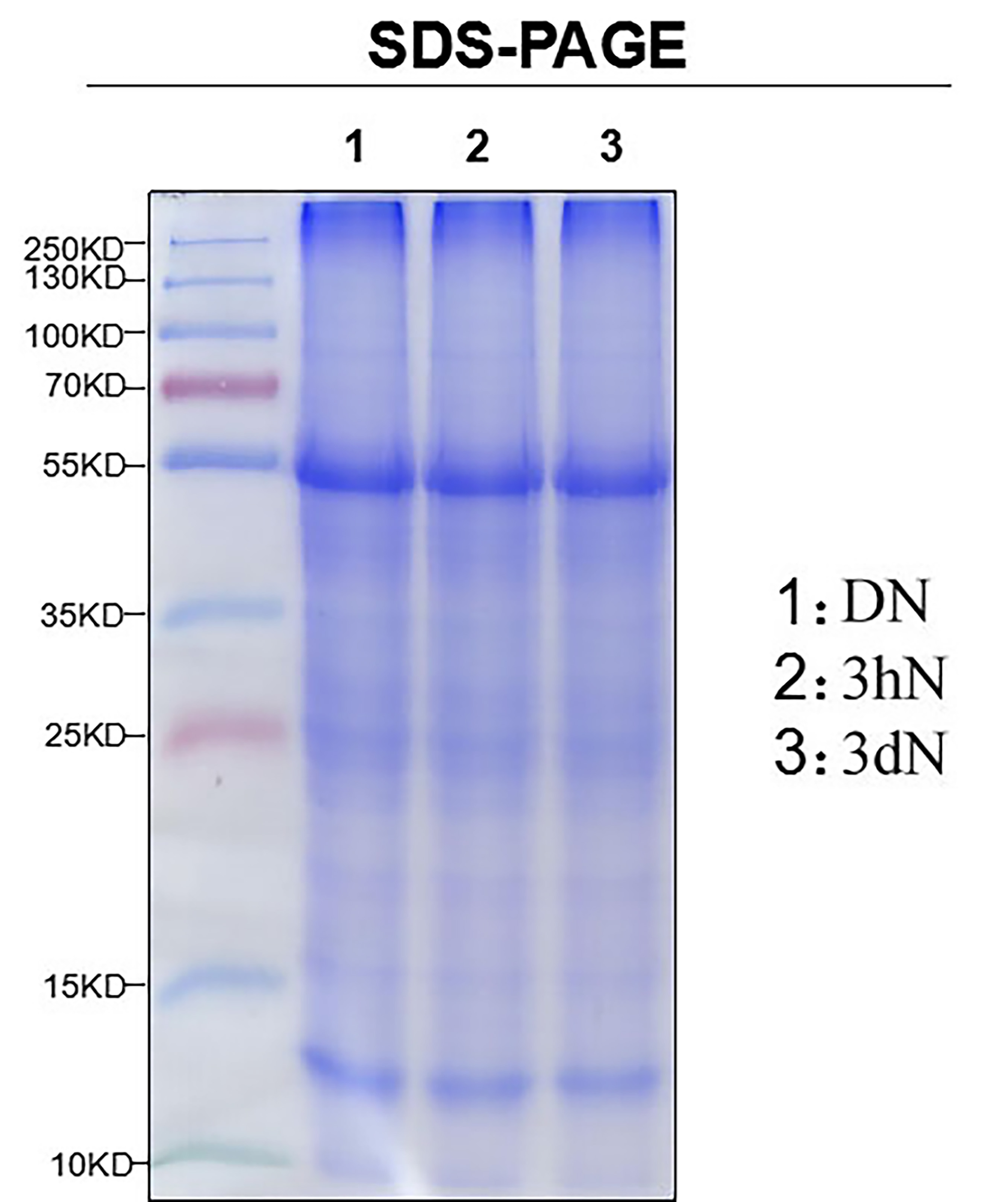


**Fig. S2** SDS-PAGE of three samples under NH_4_^+^ deficiency/resupply.


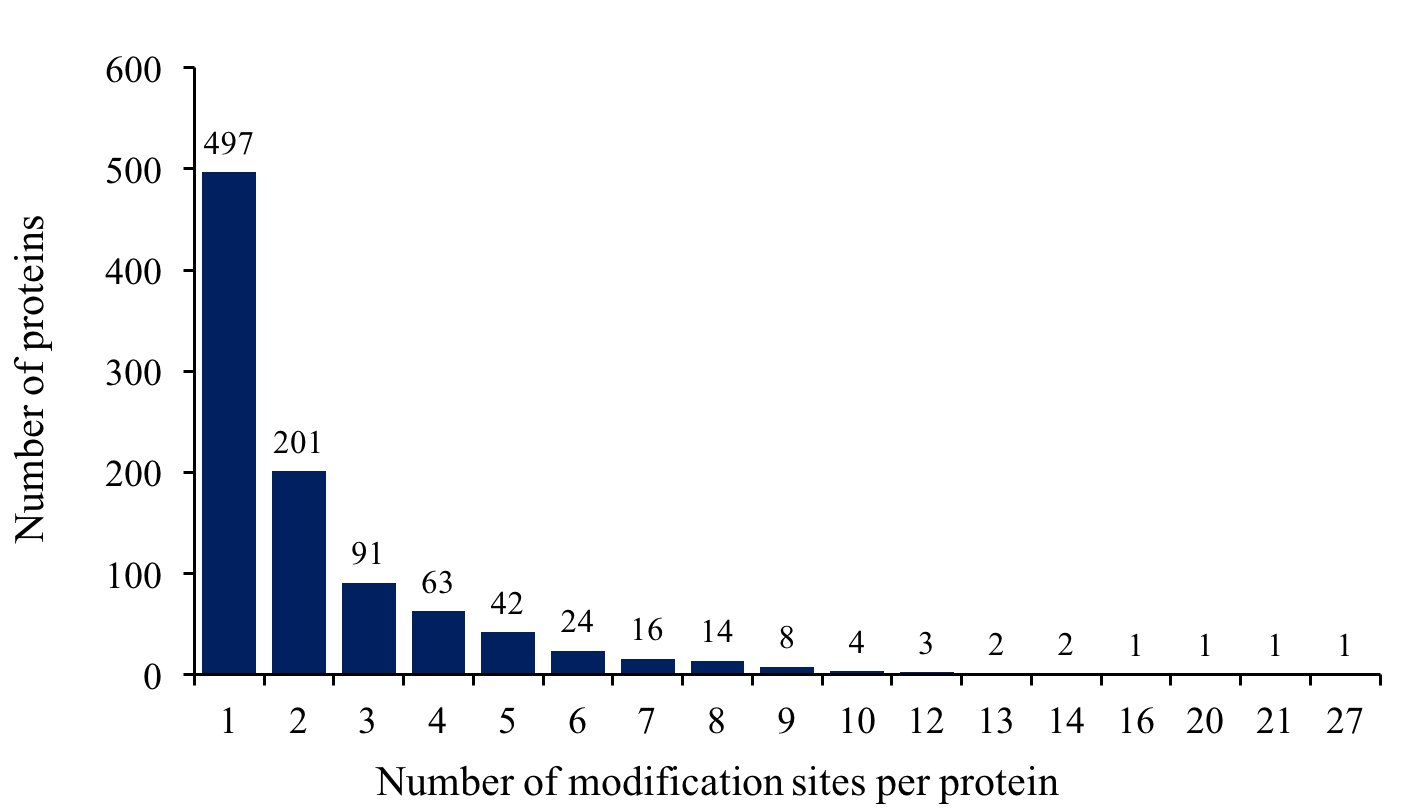


**Fig. S3** The number of Kcr sites identified per protein.


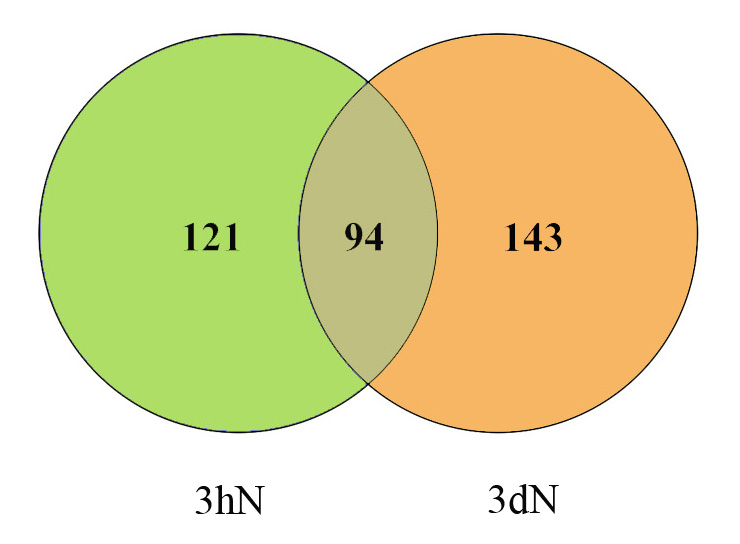


**Fig. S4** The Venn diagram analysis of DCPs at 3h and 3d of NH_4_^+^ resupply. The number of common and specific DCPs were shown in the overlapping and non-overlapping regions, respectively.
