# Supplementary material for: Ammonium triggered the response mechanism of lysine crotonylome in tea plants
Source: BMC Genomics. 2019 May 6;20:340. doi: 10.1186/s12864-019-5716-z (PMC6501322; doi:10.1186/s12864-019-5716-z)
Supplement: Supplementary file 6 — Fifty representative LC-MS/MS spectra of crotonylated peptides. (DOCX 8024 kb) [file 12864_2019_5716_MOESM6_ESM.docx]

**Fig. S1** Kcr124 on photosystem I reaction center subunit III.

**Fig. S2** Kcr121 on oxygen-evolving enhancer protein 1.

**Fig. S3** Kcr144 on oxygen-evolving enhancer protein 1.

**Fig.S4** Kcr270 on oxygen-evolving enhancer protein 1.

**Fig.S5** Kcr207 on phosphoribulokinase.

**Fig. S6** Kcr223 on phosphoribulokinase.

**Fig. S7** Kcr212 on probable ribose-5-phosphate isomerase 3.

**Fig. S8** Kcr63 on ribulose bisphosphate carboxylase small chain.

**Fig. S9** Kcr110 on ribulose bisphosphate carboxylase small chain.

**Fig. S10** Kcr138 on ribulose bisphosphate carboxylase small chain.

**Fig. S11** Kcr144 on ribulose bisphosphate carboxylase small chain.

**Fig. S12** Kcr160 on ribulose bisphosphate carboxylase small chain.

**Fig. S13** Kcr24 on fructose-bisphosphate aldolase.

**Fig. S14** Kcr116 on photosystem II Pbs27 protein.

**Fig. S15** Kcr121 on photosystem II Pbs27 protein.

**Fig. S16** Kcr169 on photosystem II Pbs27 protein.

**Fig. S17** Kcr441 on serine hydroxymethyltransferase 4.

**Fig. S18** Kcr80 on fructose-bisphosphate aldolase 1.

**Fig. S19** Kcr146 on dihydrolipoyl dehydrogenase 1.

**Fig. S20** Kcr201 on dihydrolipoyl dehydrogenase 1.

**Fig. S21** Kcr172 on fructose-bisphosphate aldolase 3.

**Fig. 22** Kcr133 on transketolase.

**Fig. S23** Kcr314 on transketolase.

**Fig. S24** Kcr480 on transketolase.

**Fig. S25** Kcr710 on transketolase.

**Fig. S26** Kcr185 on photosystem II 22 kDa protein.

**Fig. S27** Kcr117 on ATP synthase delta chain.

**Fig. S28** Kcr478 on glycine dehydrogenase.

**Fig. S29** Kcr94 on glycine dehydrogenase.

**Fig. S30** Kcr265 on glycine dehydrogenase.

**Fig. S31** Kcr84 on ribulose bisphosphate carboxylase small chain.

**Fig. S32** Kcr190 on ribulose bisphosphate carboxylase large chain.

**Fig. S33** Kcr4 on glutamate--glyoxylate aminotransferase 2-like.

**Fig. S34** Kcr35 on glutamate-glyoxylate aminotransferase 2-like.

**Fig. S35** Kcr357 on glutamate-glyoxylate aminotransferase 2-like.

**Fig. S36** Kcr81 on ferredoxin--NADP reductase.

**Fig. S37** Kcr203 on ferredoxin--NADP reductase.

**Fig. S38** Kcr238 on ferredoxin--NADP reductase.

**Fig. S39** Kcr191 on serine hydroxymethyltransferase.

**Fig. S40** Kcr314 on serine hydroxymethyltransferase.

**Fig. S41** Kcr147 on serine hydroxymethyltransferase.

**Fig. S42** Kcr237 on glyceraldehyde-3-phosphate dehydrogenase A.

**Fig. S43** Kcr194 on ATP synthase gamma chain.

**Fig. S44** Kcr125 on phosphoglycerate kinase.

**Fig. S45** Kcr119 on oxygen-evolving enhancer protein 2.

**Fig. S46** Kcr247 on oxygen-evolving enhancer protein 2.

**Fig. S47** Kcr159 on photosystem I reaction center subunit N.

**Fig. S48** Kcr108 on chloroplast sedoheptulose-1,7-bisphosphatase.

**Fig. S49** Kcr305 on chloroplast sedoheptulose-1,7-bisphosphatase.

**Fig. S50** Kcr52 on serine--glyoxylate aminotransferase.
