# Supplementary material for: Ammonium triggered the response mechanism of lysine crotonylome in tea plants
Source: BMC Genomics. 2019 May 6;20:340. doi: 10.1186/s12864-019-5716-z (PMC6501322; doi:10.1186/s12864-019-5716-z)
Supplement: Supplementary file 19 — The enzyme activities of DCPs after NH4+ resupply. (DOCX 183 kb) [file 12864_2019_5716_MOESM19_ESM.docx]

**
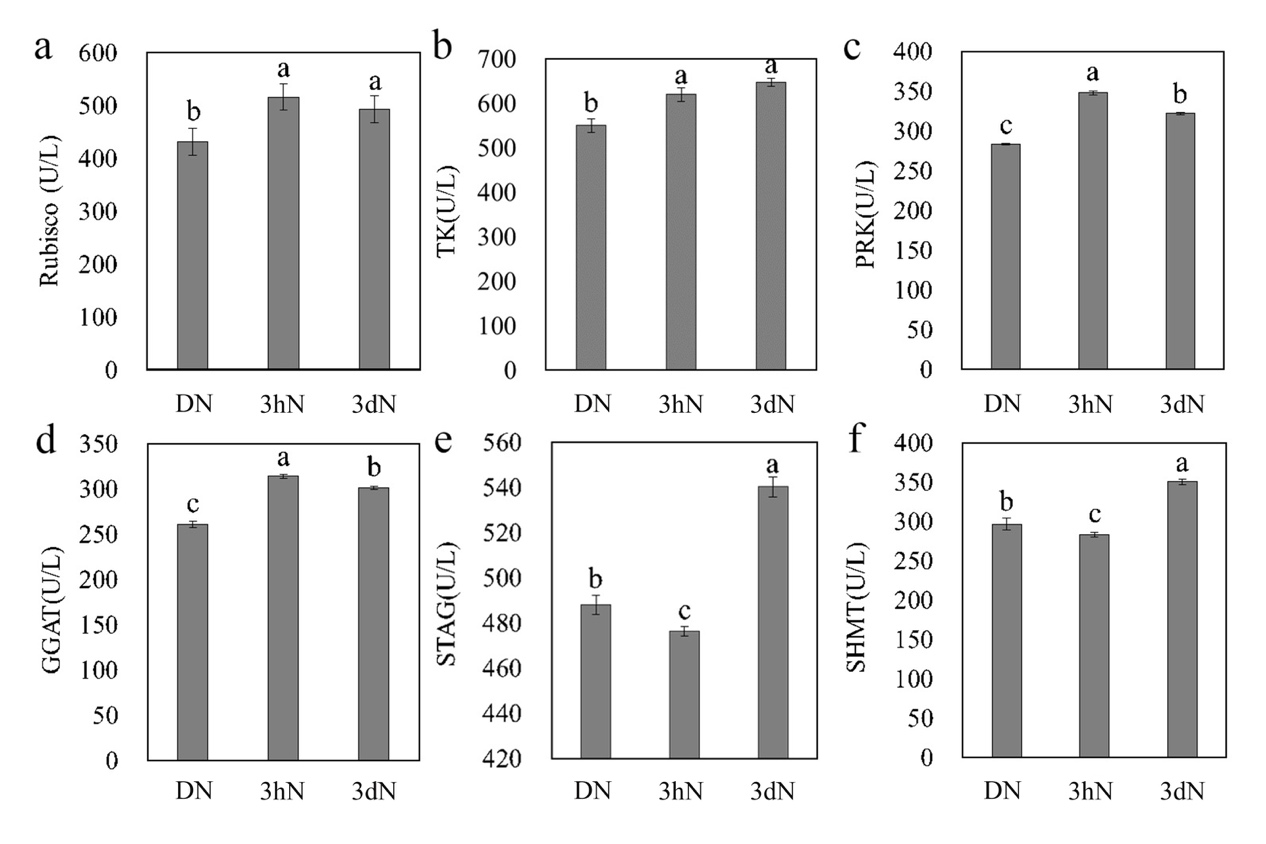
** The enzyme activities of DCPs after NH_4_^+^ resupply. **a** Rubisco activity, **b** TK activity, **c** PRK activity, **d** GGAT activity, **e** SGAT activity and **f** SGAT activity. Different letters above the bars indicate significant differences at *P* < 0.05.
